# Novel *recA*-Independent Horizontal Gene Transfer in *Escherichia coli* K-12

**DOI:** 10.1371/journal.pone.0130813

**Published:** 2015-07-10

**Authors:** Anthony W. Kingston, Chloé Roussel-Rossin, Claire Dupont, Elisabeth A. Raleigh

**Affiliations:** New England Biolabs, Ipswich, Massachusetts, 01938, United States of America; University of Massachusetts, UNITED STATES

## Abstract

In bacteria, mechanisms that incorporate DNA into a genome without strand-transfer proteins such as RecA play a major role in generating novelty by horizontal gene transfer. We describe a new illegitimate recombination event in *Escherichia coli* K-12: RecA-independent homologous replacements, with very large (megabase-length) donor patches replacing recipient DNA. A previously uncharacterized gene (*yjiP*) increases the frequency of RecA-independent replacement recombination. To show this, we used conjugal DNA transfer, combining a classical conjugation donor, HfrH, with modern genome engineering methods and whole genome sequencing analysis to enable interrogation of genetic dependence of integration mechanisms and characterization of recombination products. As in classical experiments, genomic DNA transfer begins at a unique position in the donor, entering the recipient via conjugation; antibiotic resistance markers are then used to select recombinant progeny. Different configurations of this system were used to compare known mechanisms for stable DNA incorporation, including homologous recombination, F’-plasmid formation, and genome duplication. A genome island of interest known as the immigration control region was specifically replaced in a minority of recombinants, at a frequency of 3 X 10^-12^ CFU/recipient per hour.

## Introduction

In prokaryotes, horizontal gene transfer (HGT; also called lateral gene transfer) is a massive force of evolutionary change and adaptation. It allows bacteria to adapt to ecological niches and survive in stressful conditions when traditional gene regulation is not sufficient [1]. This type of gene flow is so prevalent that it underlies many current topics in microbiology. For example, HGT is responsible for the global spread of antibiotic resistance [2,3], it shapes microbial communities like the human microbiome [4], and it is interconnected with biofilm formation [5], the CRISPR/cas immunity system [6], virulence [7,8], and other critical cellular functions. However, many aspects of HGT are still poorly understood and its overall effect on genomic evolution is the subject of active research [9].

The impact of HGT on genomic evolution is illustrated by variability of genome content within the *E*. *coli* species. In most *E*. *coli* strains, ~40% of the genome consists of variable gene islands [10,11]. These genetic elements typically encode niche-specific traits and account for the wide range of *E*. *coli* lifestyles [12]. The diversity among *E*. *coli* is so large that the pangenome, the full complement of genes discovered among *E*. *coli* strains, is estimated to be over 9 times larger than the core set of genes found in all *E*. *coli* [13]. The vast array of potential phenotypes encoded within the pangenome allows for the rapid development of new strains with niche-specific traits via genetic transfer [14,15].

For the purposes of this paper, we define HGT as the stable introduction of foreign DNA into a chromosome through a RecA-independent event [14]. This definition excludes RecA-mediated homologous recombination, which occurs at a much higher frequency than HGT. Although the literature does not always distinguish between homologous exchange and gene addition via HGT [16], we adopt a stricter definition to distinguish the distinct evolutionary consequences of the two processes. Homologous exchange tends to homogenize a population by selecting for the most favorable combination of traits within a community while HGT diversifies the population by introducing novel genetic material [17]. The two processes can complement each other. HGT can add a novel capability to one member of a population in a manner that homologous exchange cannot, and homologous exchange can then spread that capability more efficiently than the original HGT mechanisms that introduced it [14,18]. For example, a combination of HGT and homologous recombination is believed to have mediated the distribution of pathogenicity islands in *E*. *coli* that can turn a commensal strain into a virulent species [16].

A specific gene island of interest in Enterobacteriacea is known as the immigration control region (ICR). The ICR consists of a variable region of migratory genes surrounded by conserved framework genes (*yjiPRS* and *yjiAXY*) [19,20] (S1 Fig). This region shows signatures of high rates of homologous genetic exchange [14,21], but underlying that signature is a structure suggesting that it is also a likely target of site-specific replacement [22]. No mechanism for such replacement has been demonstrated.

Here we created a conjugal system to facilitate the study of RecA-independent horizontal transfer of the ICR in *E*. *coli* and relatives, and qualified its properties in the model strain K-12. This conjugal system proved to be an effective tool for discovering and analyzing novel mechanisms of horizontal gene transfer. Although it was designed to focus on a specific gene island (the ICR), it revealed a previously unreported capacity for large-chunk exchange in the absence of RecA. Most recombinants obtained arise from the replacement of large (0.1–2.4 Mb) segments of genomic DNA; about 1.4% have replaced only the ICR (~16 kb). We also identified the previously uncharacterized protein YjiP as an enhancer of *recA*-independent recombination.

## Experimental Procedures

### Strains, plasmids, and growth conditions

All strains, plasmids, and oligonucleotides used in this study are listed in S1 Table. Bacteria were grown in liquid Luria Broth (LB; 10 g/L Tryptone, 5 g/L yeast, 10 g/L NaCl) or rich broth (RB; 10 g/L Tryptone, 5 g/L yeast, 5 g/L NaCl, pH 7.2) medium at 37°C with vigorous shaking or on solid LB or RB medium containing 1.5% agar with appropriate selection. Ampicillin (Ap; 100 μg/mL) streptomycin (Sm; 100 μg/mL), kanamycin (Kn; 40 μg/mL), chloramphenicol (Cm; 30 μg/mL), tetracycline (Tc; 20 μg/mL), and nalidixic acid (Nl; 5 ug/mL in Δ*recA* strains and 20 ug/mL in *recA* ^+^ strains) were used to select *E*. *coli* transformants. Plasmids were prepared from *E*. *coli* NEB Turbo cells. For temperature sensitive plasmids, 30°C incubation was used to permit plasmid replication and 42°C incubation was used to remove the plasmid.

### Genetic and molecular techniques

Linear and plasmid DNA transformations were performed as described previously [23] as were transductions using the *P1vir* bacteriophage [24]. DNA constructs were created using the Gibson assembly kit (#E2611; New England Biolabs). Chromosomal gene deletions were generated using the λ Red recombinase system [25]. Unless otherwise stated, all PCR products were generated using *E*. *coli* MG1655 chromosomal DNA as a template and all strains were verified by PCR and/or sequence analysis (New England Biolabs DNA sequencing facility). PCR reactions used to generate sequencing templates or genetic constructions were performed with the Q5 High-Fidelity DNA Polymerase (#M0491; New England Biolabs) while diagnostic PCR reactions used the Hot Start Taq 2X Master Mix (#M0496; New England Biolabs). New England Biolabs produced all restriction enzymes and ligases used in these experiments.

### Matings

Donor and recipient cultures were grown at 37°C to an OD_600_ of ~1.0 in RB with shaking. Mating was initiated by mixing the cultures in a 1:1 ratio. To ensure optimal stability of the F pilus, the mating mixtures were aerated with slow rolling in a tube roller. The duration of the mating was 0.25 h when the strains involved contained functional *recA* or *repE* genes because recombination efficiency was high. When the strains lacked both *recA* and *repE*, recombination efficiency was much lower, so mating duration was increased to 18 hours to yield sufficient recombinants. For rhamnose induction, rhamnose was added to recipients 3 hours before the start of the mating (when the mating duration was 15 minutes) or at the start of mating (when mating duration was 18 hours).

After mating, cells were diluted up to 10^-6^ and plated on antibiotic media selective for donors, for recipients, and for recombinants at dilutions that would yield at least 10–100 colonies per plate or as much as remained for recombinants. In matings with low recombination efficiency, up to 6 mL of the mating culture was centrifuged, re-suspended in residual broth, and spread out over multiple recombinant selective plates to obtain enough recombinants (1 mL culture per plate). The strain combinations, mating durations, and concentrations of antibiotics used for selection in all matings are described in S2 Table. Plates were incubated for 48h at 37°C and counted to calculate recombination efficiency and cell survival. Unmated donor and recipient cultures were subject to identical procedures to determine CFU/mL levels in unmated cells and to confirm that spontaneous mutants do not appear.

### Characterization of recombinants

Recombinant colonies were purified once on the same selection media and tested for antibiotic resistance and UV sensitivity.

#### Antibiotic screens

Recombinants were streaked onto RB plates supplemented with kanamycin or chloramphenicol and incubated at 37°C. Strains that failed to grow or exhibited only sporadic colonies arising after 48 h were classified as sensitive to that antibiotic.

#### UV sensitivity screens

Recombinants were streaked horizontally across an RB plate alongside control *rec* ^+^ and *rec* ^-^ strains. Vertical portions of the plate were exposed to UV light (380 nm) for 0 to 60 seconds, then incubated in the dark for 24 h. Recombinants were classified as UV sensitive if they behaved similarly to the *rec* ^-^ control with only scattered colonies surviving at any exposure or as UV resistant if growth was confluent at UV exposure under 30 seconds with minor cell death at higher exposure times (as for the *rec* ^+^ control).

### Testing the effect of rhamnose induction on cell viability

Cultures were grown to an OD_600_ of ~0.2 in RB and then split into multiple tubes. Cultures were treated with rhamnose concentrations ranging from 0–0.2% using a stock 20% L-rhamnose solution. Cells were grown at 37°C with constant shaking at high aeration levels for 3 hours. Every 30 minutes, a small aliquot of cells was diluted up to 10^-6^ and plated on streptomycin media. At 3 hours after rhamnose treatment, cells were split again and either mated with an equivalent volume of the donor strain ER3435 grown alongside the recipient or left untreated. Aliquots were plated after mating on streptomycin media at 30 minutes and 18 hours. Plates were incubated for 24h at 37°C and counted to determine cell survival. The CFU count for mated cells was doubled to account for dilution with the donor strain.

### β-galactosidase assays

See supplemental methods (S1 File).

### Genomic Sequencing

#### Illumina Sequencing

A detailed description of the Illumina Sequencing process can be found in the supplemental methods (S1 File).

#### Pacific Biosciences SMRT Sequencing

Genomic DNA samples were prepared for SMRT sequencing as previously described [26]. Briefly, genomic DNA from an overnight recombinant culture was purified using standard phenol-chloroform extraction methods and quantitated using a Qubit 2.0 Fluorometer (Life Technologies, Grand Island, NY, USA). DNA samples were sheared to an average size of ~10 kb using Covaris G-tubes (Covaris, Woburn, MA, USA). Further DNA purification was achieved with MO BIO PowerClean DNA Clean-Up Kit (MO BIO Laboratories, Carlsbad, CA, USA) and AxyPrep Mag PCR Clean-up kit (Axygen, Union City, CA, USA). SMRTbell template libraries were prepared using the 10 kb preparation protocols provided by Pacific Biosciences (http://www.pacificbiosciences.com/samplenet/ProcedureChecklistLowInput10kbLibraryPreparationandSequencingMagBeadStation.pdf) and the reagents supplied in the SMRTbell Template Prep Kit 1.0 for DNA repair, ligation, and exonuclease digestion (Pacific Biosciences, Menlo Park, CA, USA). Products were verified for size using the Agilent 2100 Bioanalyzer and DNA 12000 Kits (Agilent Technologies, Santa Clara, CA, USA). Polymerase binding was accomplished with the DNA Polymerase Binding Kit 4.0, Magbeads, and the Magbead Buffer Kit (Pacific Biosciences, Menlo Park, CA, USA). Libraries were sequenced with the DNA Sequencing Kit 3.0 on a PacBio RS instrument and reads were analyzed with the Pacific Biosciences SMRT analysis software v2.2.0.

#### Variant visualization using Geneious

De novo assembled contigs were imported into Geneious 7.1.7 (Biomatters Ltd) and annotated. Edges were trimmed and the sequence was circularized to obtain a complete genome. Nucleotide #1 was set as the *dnaA* translation start site. Pairwise alignments of the two parents with each other and with each recombinant were made using Mauve and variants were visualized for each comparison. A more detailed explanation of this process can be found in the supplemental methods (S1 File). The annotated sequences are on deposit at NCBI: http://www.ncbi.nlm.nih.gov/bioproject/271807

Accession: PRJNA271807 ID: 271807

### Statistical analysis

All experiments were performed with a minimum of three biological replicates. Unless otherwise noted, data is presented as mean ± standard error. Unpaired Student's *t* tests were used for statistical evaluation. A value of *P* ≤ 0.05 was considered statistically significant.

## Results

### The conjugal system

**Fig 1:**
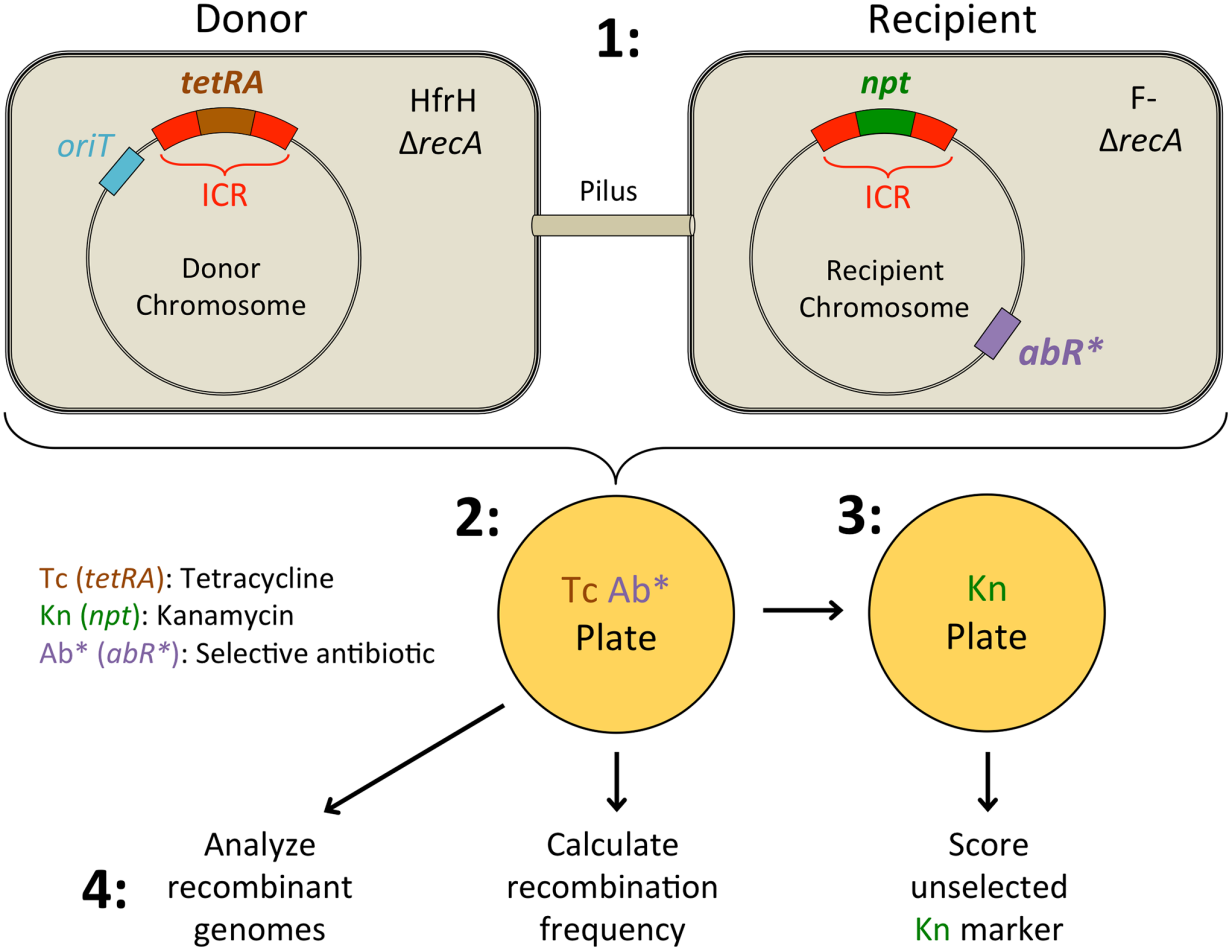
Model system for studying recombination events involving the ICR. 1: DNA transfer is mediated by a conjugal mating between an HfrH donor and a F^-^ recipient. Transfer begins at *oriT* and proceeds in the direction of the nearby ICR. Both strains are Δ*recA* to prevent homologous recombination. The donor contains a tetracycline resistance regulon (*tetRA*) within the ICR. The recipient carries unselected scoreable drug markers within the ICR, and counter-selection markers (resistance to streptomycin, nalidixic acid, and/or chloramphenicol) elsewhere (AbR*). During the mating, leading-strand DNA synthesis starting at *oriT* in donor displaces the complementary single strand, which is transferred to the recipient, where lagging-strand synthesis copies it. Transfer begins with *oriT* and moves clockwise through the *tetRA*-ICR construct. 2: Cells are plated on Tet + Ab* media, selecting for recombinants that have received a stable copy of *tetRA* in a recipient background. 3: Recombinants are tested for the unselected marker, kanamycin sensitivity, to see if the incorporated *tetRA* replaced *npt*). 4: In some experiments, parents and recombinants were analyzed by sequencing: Whole genomes were assembled and annotated, then aligned for identification of 450 segregating variant positions.

We designed a system that will efficiently introduce a donor copy of a variable gene island, the ICR, into a recipient cell with a different version of it (Fig 1, Table 1).

**Table 1:** Relevant features of the donor and recipient strains in the mating system.

Donor Strain		Recipient Strain	
Genotype	Function	Genotype	Function
*oriT* (HfrH)	Transfer of donor genomic DNA	F-	Cell can receive F DNA
*traI-traM*	F pilus and transfer replicon	*recD1014*	Stabilize Invading DNA
Δ*recA*	Prevent homologous recombination	Δ*recA*	Prevent homologous recombination
ΔrepE*^a^	Prevent F plasmid formation	abR*^b^	Select for recipient cells
ICR*(mrr)*::*tetAR*	Select for donor DNA integration	ICR*(yjiT-mrr)*::*npt* or yjiP::cat yjiT::npt^c^	Unselected markers; Screen for ICR replacement

^a^ This mutation deletes the entire proximal F DNA segment from the donor strain, but if is referred to as ΔrepE* (rather than Δ(*ygfA-ycaA*)::(FRT)) for simplicity.

^b^abR* refers to resistance markers for streptomycin (SmR; *rpsL104*), chloramphenicol (CmR; *recA*::*cat(FRT)*), and/or nalidixic acid (NlR; *gyrA96*)

^c^In crosses 1–6 and 11–13, genotype is ICR*(yjiT-mrr)*::*npt*. In crosses 7–10 the genotype is *yjiP*::*cat*(FRT) *yjiT*::*npt*(FRT). See Fig 2 for a depiction of these genotypes.

#### The Donor

**Fig 2:**
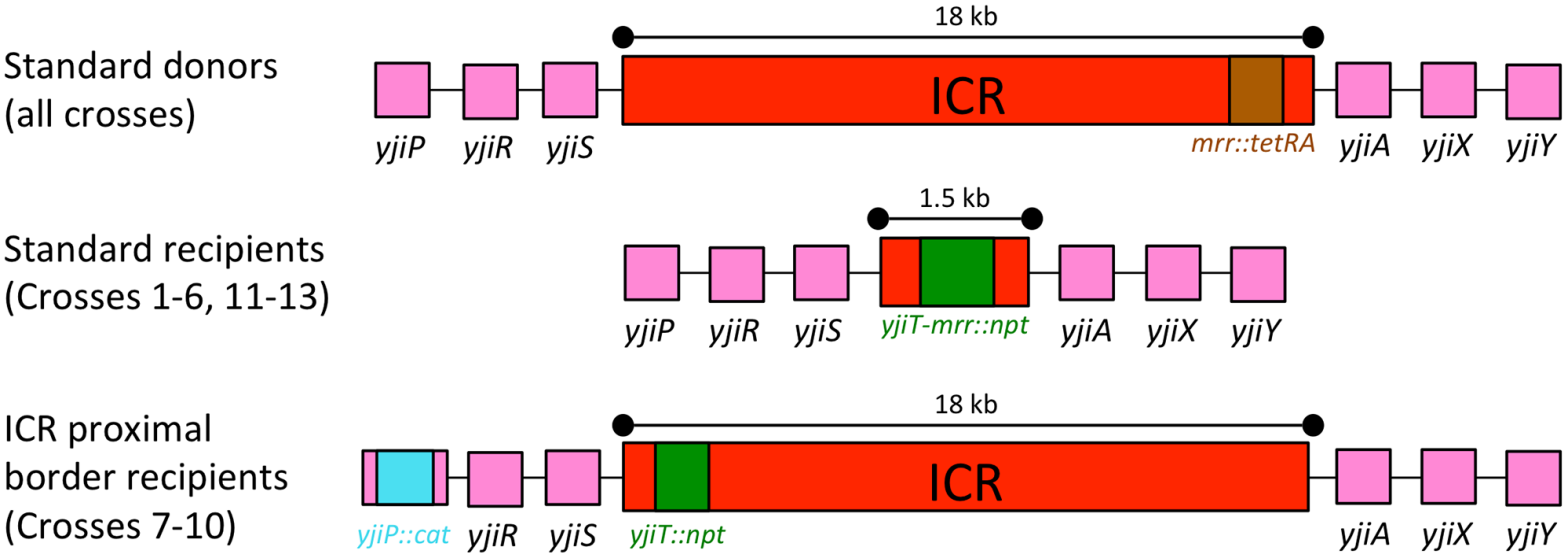
The ICR region in the standard donors (ER3276 & ER3435), standard recipients (ER3436 & ER3460) and ICR proximal border recipients (ER3472, ER3473, ER3480, & ER3481) (not to scale).

This system relies on Hfr conjugal transfer of the chromosome. A conjugal plasmid, the F (fertility) plasmid, is integrated near the locus of interest. DNA transfer begins at *oriT* within the F and proceeds in one direction (here, towards the adjacent ICR) at about 40 kb/min [27]. The entire donor genome is transferred in 100 min [28]. Transfer is rapid and efficient: most recipients in laboratory matings make contact with a donor within minutes [29] and subsequent DNA transfer occurs almost immediately [30]. In the HfrH strain we began with, *oriT* is ~250 kb from the ICR, ensuring that recipients will receive the donor ICR less than 15 minutes after mating is initiated. To prevent transfer of a wild-type copy of *recA*, the coding sequence was deleted from the donor as well. Selection for the donor ICR was enabled with an added tetracycline resistance marker (TcR; *mrr*::*tetAtetR*) (Fig 2). This disrupts a gene that is not a candidate for participation in recombination events.

#### Recipients

The recipient ICR was treated as an unselected marker. In initial experiments, a kanamycin resistance cassette (KnR; Δ(*yjiT*-*mrr)*::*npt(FRT)*) replaces the entire ICR (Fig 2). Some of the later experiments involved recipients in which kanamycin and chloramphenicol resistance cassettes flank the proximal ICR border. In all recipients, distant drug resistance markers streptomycin (SmR; *rpsL104*), chloramphenicol (CmR; *recA*::*cat(FRT)*), and/or nalidixic acid (NlR; *gyrA96*) were used for counterselection. S2 Table lists antibiotic combinations used for selection in all matings. To stabilize the donor substrate in the recipient cell, the Exonuclease V activity of RecBCD was inactivated with a *recD1014* mutation. This mutation increases recombination efficiency in RecA^+^ assays [30–33]. The (Δ*recA*::*cat(FRT*)) marker removed *recA* from the recipient.

#### Validation experiments comparing the conjugal system

**Fig 3:**
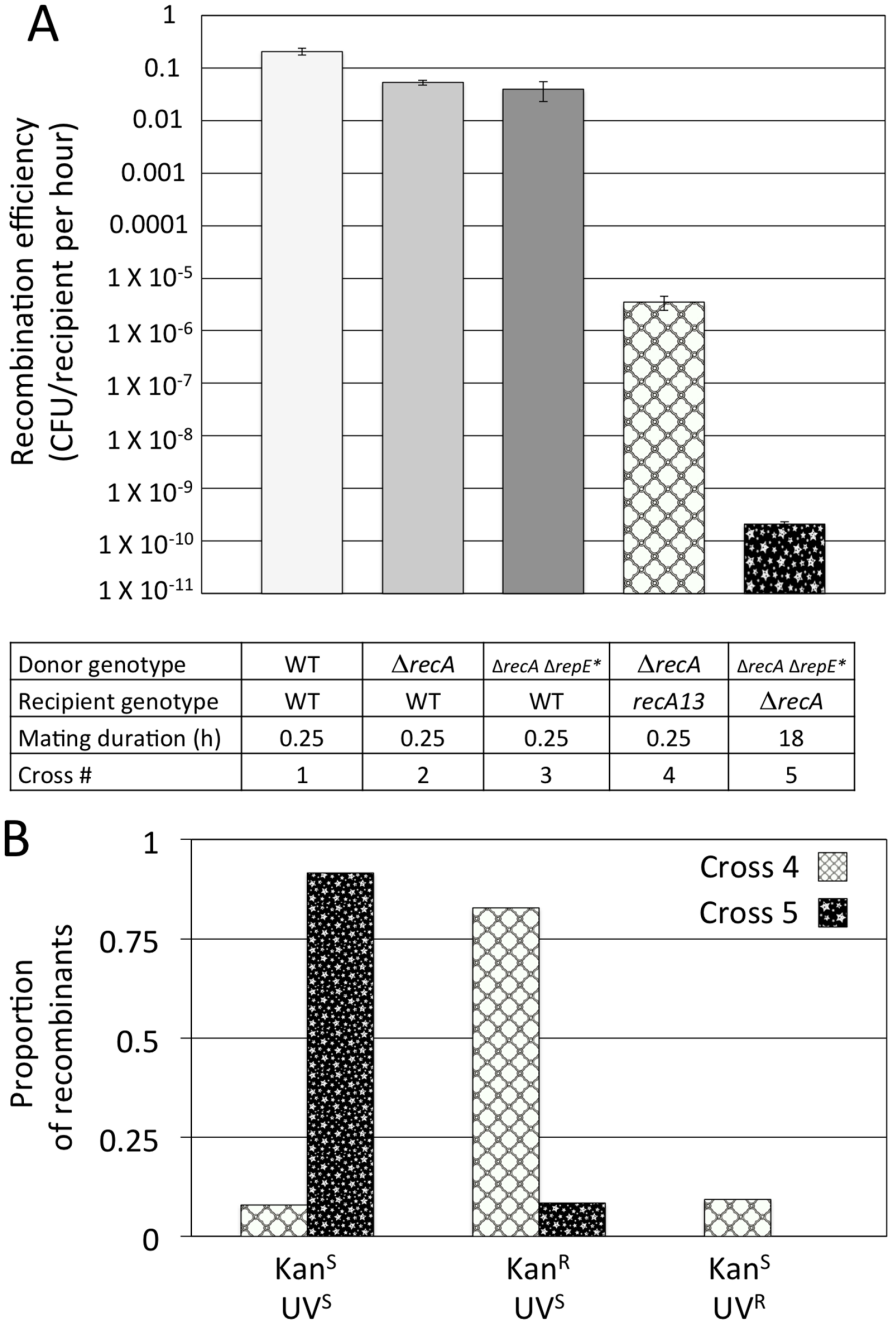
The Effect of *recA* and *repE* on the mating system. (A) The recombination efficiency per recipient per hour in mating crosses: 1) WT donor (ER3270) X WT recipient (ER1636), 2) Δ*recA* donor (ER3276) X WT recipient (ER1636), 3) Δ*recA* ΔrepE* donor (ER3435) X WT recipient (ER1636), 4) Δ*recA* donor (ER3276) X *recA* recipient (ER3263), and 5) Δ*recA* ΔrepE* donor (ER3435) X Δ*recA* recipient (ER3436). See S2 Table for more details on individual crosses. The legend indicates status of *recA* and *repE* in the donor and recipient, mating duration, and which mating pair was used in each cross. Recombination efficiency is reduced by deleting *recA* from either the donor or recipient to eliminate homologous recombination, and with the ΔrepE* deletion in the donor to prevent F’-plasmid replication. All crosses were performed with a minimum of three biological replicates. Error bars represent standard error. (B) The proportion of recombinants that are kanamycin sensitive (KnS), kanamycin resistant (KnR), or UV resistant (UV^R^) from cross (4) Δ*recA* donor X *recA13* recipient and from cross (5) Δ*recA* ΔrepE* donor X Δ*recA* recipient. Most recombinants were KnR when the donor had *repE* in the leading F DNA because RepE allows for stabilization of donor DNA by plasmid replication (S3 Fig) [34]. In the absence of plasmid formation, crosses resulted primarily in KnS recombinants. In cross (4), UV^R^ recombinants resulted from reversion of the ER3263 recipient’s *recA13* point mutation allele [35,36]. To avoid this we deleted *recA* from recipient ER3436 for cross 5.

The plasticity of our conjugal system allows us to create multiple configurations that are each designed to study a different type of recombination event. This analysis provides us with a way to estimate the relative frequency of different mechanisms of genomic evolution. A complete analysis of the configurations tested can be found in S2 File, but a brief overview is provided here. The high efficiency of the system was confirmed with a RecA^+^ configuration in which ~20% of the recipients acquired tetracycline resistance from the donor per hour of mating (Fig 3A). Inactivating RecA reduced recombination efficiency ~10000 fold, but most recombinants from this configuration were created by the formation of novel F’-plasmids (S2 Fig). To suppress formation of these F' derivatives we deleted *repE* which is required for extrachromosomal vegetative replication [34] (S3 Fig). This further reduced mating efficiency to a low but measurable (2.1 ± 0.2) X 10^-10^ CFU/recipient per hour. Most recombinants were KnS, suggesting RecA-independent genomic replacement (Fig 3B).

#### Regulated overproduction of candidate participant *yjiP*

After validating the system, we used it to test a candidate participant in RecA-independent genomic replacement. This was *yjiP*, an uncharacterized gene near the proximal border of the ICR (Fig 2). YjiP was hypothesized to contribute to site specific replacement at the ICR because it is highly conserved compared to the ICR contents [22], and it contains a PD-(D/E)XK phosphodiesterase domain suggesting nuclease activity [37]. Most *E*. *coli* strains encode a complete YjiP protein, but *yjiP* from *E*. *coli* K-12 (which our recipients are derived from) contains a point mutation that results in a premature stop codon. We synthesized a mutated K-12 *yjiP* lacking the premature stop codon (*yjiPc*, for *yjiPcomplete*), fused it to the rhamnose inducible promoter *rhaBp*, and integrated this *rhaBp-yjiPc* construct into the Tn7 attachment site of recipient strains [38]. The *rhaBp* promoter is essentially silent in the absence of rhamnose, and inducible 200-fold in its presence (S4A Fig).

### A functional role for YjiP

#### YjiP expression increases the frequency of RecA-independent recombination

**Fig 4:**
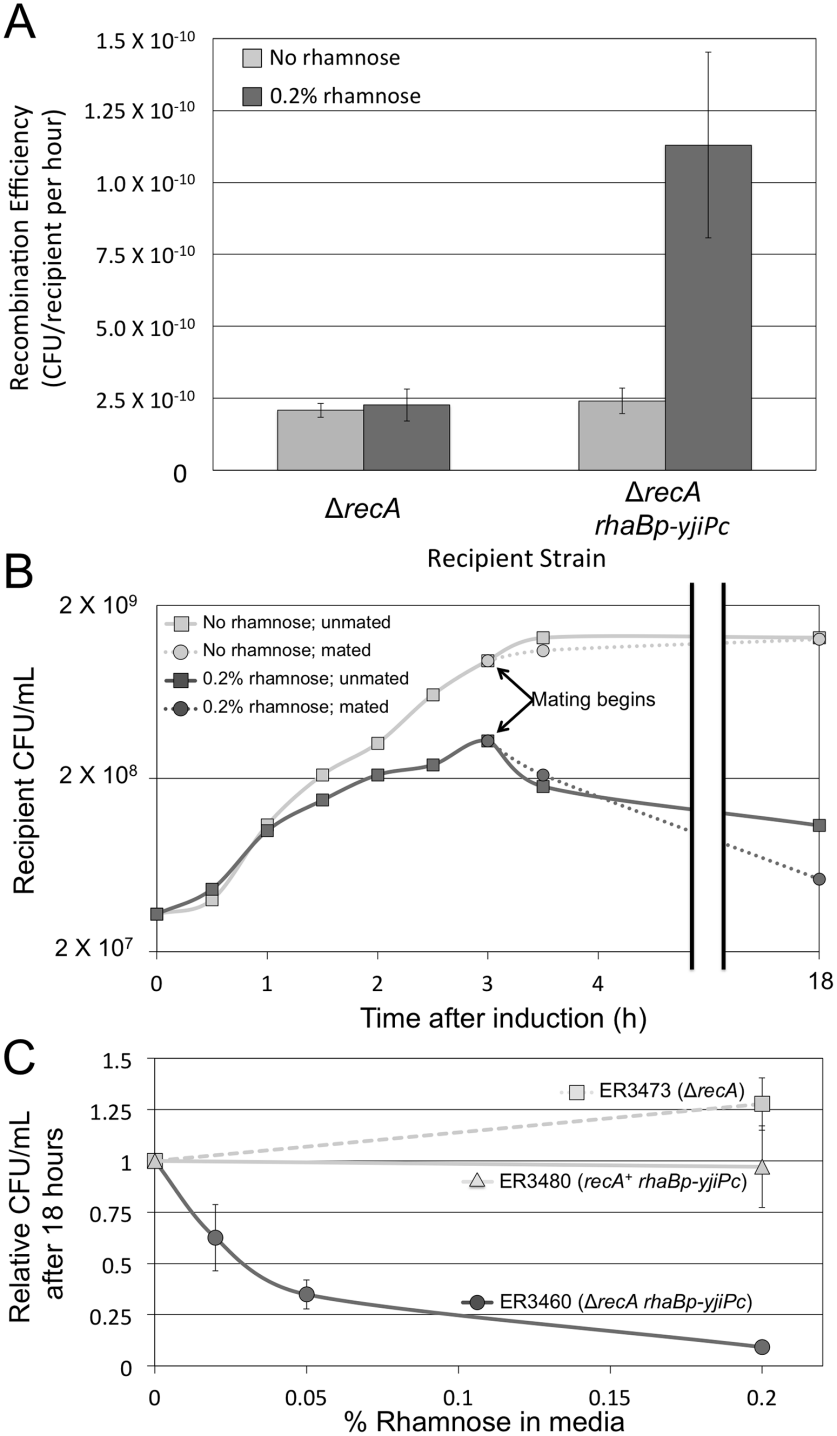
YjiP promotes *recA*-independent recombination and reduces cell viability. (A) The frequency of recombination during matings between the ΔrepE* Δ*recA* donor and either the Δ*recA* recipient (cross 6; Δ*recA*) or a Δ*recA* recipient with inducible overexpression of *yjiP* (cross 13; Δ*recA rhaBp-yjiPc*), with and without 0.2% rhamnose. Recombination efficiency was calculated as the frequency of recombinant formation per viable recipient per hour in the mating mixture. Inducing *yjiPc* expression with rhamnose significantly increases recombination efficiency ~5 fold (P-value = 0.018), but rhamnose has no significant effect when the recipient lacks the *rhaBp-yjiPc* gene fusion. (B) Cell viability over time of the Δ*recA rhaBp-yjiPc* recipient (ER3460) grown in 0.2% rhamnose. This experiment was performed three times with biological replicates, but only the results of a single representative trial are shown for clarity. Rhamnose was added at t = 0, and cells were mated after 3 hours of growth with ER3435. Untreated and unmated cells were also included as controls. Rhamnose-induced *yjiPc* expression reduces cell proliferation for the first three hours after treatment and begins to kill cells afterwards. Mating did not significantly affect cell viability. (C) Dose-response of cell killing: fraction starting titer for three strains at 18 hours as a function of inducer concentration. Strains were Δ*recA* (ER3473), *recA* ^+^ *rhaBp-yjiPc* (ER3480), and Δ*recA rhaBp-yjiPc* (ER3460) grown in various concentrations of rhamnose for 18 hours relative to an untreated control. Higher concentrations of rhamnose are increasingly lethal to ER3460; 0.2% rhamnose kills ~90% of the normally viable cells. All experiments in panels A and C were performed with a minimum of three biological replicates with error bars representing standard error.

RecA-independent recombination efficiency increased significantly with increased *yjiPc* expression in the recipient. In a mating between the Δ*recA rhaBp-yjiPc* recipient and the Δ*recA* ΔrepE* donor (Cross 13), rhamnose addition increased recombination efficiency over 5 fold (Fig 4A). A similar increase in recombination efficiency upon *yjiPc* induction was observed when mating with a Δ*recA repE* ^+^ donor, but *yjiPc* induction had no effect on recombination efficiency in a *recA* ^+^ recipient (Crosses 12 & 9 respectively, S4B and S4C Fig). A different ICR framework gene, *yjiA*, with a *rhaBp-yjiA* construct (ER3333), had no effect on recombination efficiency (Cross 11; S4C Fig). Thus, we conclude that *yjiPc* expression specifically increases the frequency of *recA*-independent recombination in our conjugal system.

#### YjiP expression reduces viability

In addition to increasing recombination efficiency, *yjiPc* expression reduced cell viability in Δ*recA* recipients. The addition of 0.2% rhamnose, to the Δ*recA rhaBp-yjiPc* recipient (ER3460) reduced the growth rate between 0–3 hours after treatment (as measured by CFU/mL on selective media) (Fig 4B). Cells began to die after 3 hours. At 3 hours, a surrogate reporter of expression potential (β-galactosidase) has reached a maximum (S4A Fig). By 18 hours CFU count was over 10 fold lower than an uninduced control. Cell death is dose-dependent when *yjiPc* is expressed; lower concentrations of rhamnose improved cell viability (Fig 4C) for the overexpresser, ER3460. Rhamnose did not significantly affect cell viability in a Δ*recA* background without inducible *yjiPc* (ER3473) or when *yjiPc* is overexpressed in a *recA* ^+^ background (ER3480)

The mechanism by which *yjiPc* reduces cell viability is unclear, but clues suggest that DNA damage may occur. The non-viable cells do not appear to be lysing, as OD_600_ readings of a rhamnose treated culture are indistinguishable from a non-treated control (S4D Fig). The introduction of foreign DNA did not trigger cell death either: mating the *yjiPc*-expressing recipients with a donor strain did not immediately affect cell viability (Fig 4B). Mating itself may exacerbate this effect, as viability is reduced at the 18 hour timepoint.

We confirmed that yjiP overexpression results in DNA damage by testing its effect on the SOS response. A plasmid containing the rhamnose inducible yjiP construct was introduced into a *rec* ^+^ strain in which *lacZ* is fused to the DNA damage inducible *dinD* locus on the E. coli genome (*dinDp-lacZ; [39]*). Growing this strain (ER3544) on X-gal plates showed that the presence of rhamnose significantly increased *lacZ* expression (S4E Fig). Thus, we concluded that *yjiP* expression can cause enough DNA damage to result in an SOS response.

### RecA-independent recombinants have replaced large segments of the recipient genome with the donor copy

#### Sequence characterization of recombinants

**Fig 5:**
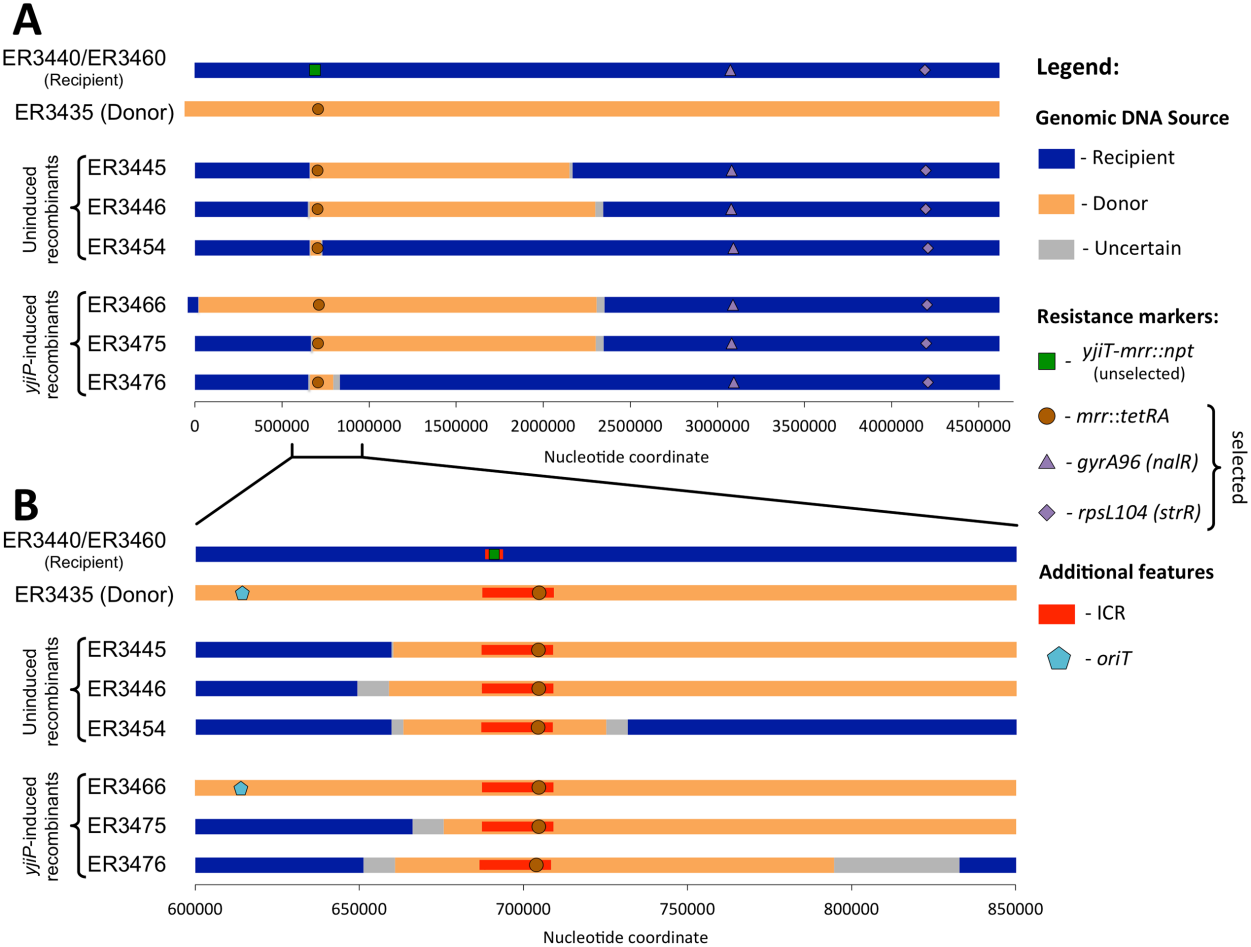
Long Patch Replacements occur in RecA-independent crosses. We compare (A) the entire genome and (B) the genomic region surrounding the ICR between the ΔrepE* Δ*recA* donor (ER3435), the Δ*recA*/Δ*recA rhaBp-yjiPc* recipients (ER3440/ER3460), and six recombinants produced by mating these strains (Cross 6; uninduced recombinants, and Cross 13; *yjiPc*-induced recombinants). Each bar represents the genome for a single strain with the color indicating the origin of genomic DNA. See S5 Fig or an explanation of how the parent was identified for each position and the schematic created. Each recombinant exhibits a unique recombination patch that extends beyond the borders of the ICR. In four of the six cases, over 1.5 megabase of contiguous donor DNA replaced the corresponding recipient DNA. All markers are positioned to align with the common genomic backbone (i.e excluding indel variants such as F insertion). Coordinate 1 is placed at the translation start site of *dnaA* instead of the traditional coordinate #1 upstream of *thrA*.

Variations between the donor and recipient genomes allowed us to characterize the genomic exchange events that created several Δ*recA* recombinants. Although the donors and recipients are both K-12 descendants, the two lineages separated in the early 1950's and vary at about 450 positions, counting each insertion or deletion as a single position (about 1 variation per 10000 bp (S5 Fig Box 1)). We selected three uninduced KnS recombinants from Cross 5 and three *yjiPc*-induced KnS recombinants from Cross 13 for long-read Pac Bio SMRT sequencing and genome alignment (Fig 5). All recombinant genomes (uninduced: ER3445, ER3446, & ER3454; induced: ER3466, ER3475, & ER3476), along with the donor (ER3435) and recipient (ER3436) genomes were successfully sequenced and assembled *de novo*. Entire genomes were then aligned with Mauve [40] as implemented in the *Geneious* program package. The Genious alignment view enabled visualization of variant positions in the recombinant genomes and their assignment to recipient and donor (S5 Fig Boxes 2–4). The results are shown schematically in Fig 5.

This analysis revealed that all six recombinants were created by large replacements of recipient genomic DNA with the corresponding genomic DNA from the donor (Fig 5). The exchanged DNA always included the ICR, as demanded by selection for the antibiotic resistance marker. However, we were surprised to find that each exchange event replaced a unique segment of DNA that extended well beyond the borders of the ICR. In four of the recombinants, >1.5 Mb of donor DNA replaced the corresponding DNA on the recipient genome. The other two recombinants replaced less than 200 kb of DNA.

To understand how these large patch replacement events could occur in a RecA-deficient background, we focused our attention to the edges of DNA exchange in the six recombinants, which we refer to as crossover intervals. In this context, each recombinant results from 2 distinct crossover events: a proximal crossover in which the recipient genome connects to a segment of donor DNA proximal to the ICR, and a distal crossover in which that segment of donor DNA links to the recipient genome distal to the ICR. Because of the low density of informative variant positions distinguishing donor from recipient, these crossover intervals ranged from 0.5 to 50 kb.

The proximal crossover intervals are each different, but show some evidence of nonrandom distribution. The proximal crossover will be partially constrained to occur in the ~75 kb of chromosomal DNA between *oriT* and the ICR, since conjugal DNA transfer begins at *oriT* and proceeds in the direction of the ICR. Five of the six proximal crossovers occur in a 26 kb region between *oriT* and the ICR, which is only one third the size of the available segment (S6 Fig). The proximal crossover intervals for the three uninduced recombinants were particularly close together, spanning only 16.6 kb and overlapping each other. Although the number is small, this clustering suggests regional preference for the proximal crossover. In the sixth (*yjiPc*-induced) recombinant (ER3466) the proximal crossover occurred between the chromosomal replication origin and *oriT*. This can only occur if the donor transfers its entire chromosome, before transferring the ICR a second time via rolling-circle replication in the donor [41].

The distribution of distal crossovers also suggests a potential recombination hotspot. Three of the six crossovers (one uninduced and two *yjiPc*-induced) occurred in the same 44.5 kb interval located over 1.5 Mb away from the ICR. Since the distal crossover in this mating system could occur anywhere in the ~2.4 Mb between the ICR and the *gyrA96* antibiotic resistance marker, the fact that 50% of the pooled set of sequenced recombinants show crossover in the same 44.5 kb (2% of available sequence) suggests a nonrandom event. We attribute this to the action of the XerCD recombinase at the *dif* site found in this interval (see Discussion).

We list the genes and features found in the crossover intervals observed in S3 Table. Some interesting ones associated with mobile elements, defining boundaries for all six recombinants, are briefly described here. The *fimE*/IS1 informative marker defines an edge of the proximal crossover interval in three recombinants, two uninduced (ER3445 & ER3454) and one *yjiP*-induced (ER3476). This marker consists of a copy of the IS1 mobile element that has inserted into the *fimE* gene in the recipient, but not in the donor (S6 Fig) [9]. An IS element variant, *yjiC*/IS5, also defines an edge of a fourth proximal crossover interval, for ER3475: an IS5 element has integrated in to *yjiC* in the recipient, but not in the donor [42]. A third marker associated with DNA mobility, and defining an edge of the proximal crossover for two recombinants (ER3445 and ER3454), is the invertible segment *fimS* [43,44]. This segment is oriented differently in the donor and the recipient. One of these recombinants (ER3454) exhibits a unique *fimS* sequence compared to both the donor and recipient. An interpretation of how these genetic elements may contribute to the recombination events can be found in the discussion.

The genome of one KnR recombinant from Cross 5 (ER3455) was also sequenced. Mapping reads from the sequencing against either the donor or recipient strains showed a large region of double coverage between the *rrlE* and *rrsH* ribosomal RNA genes (S7 Fig). Genome duplications are frequent in *E*. *coli*, especially between repetitive sequences like ribosomal operons [45], so we explored the possibility that such a duplication occurred. Using the Pacific Biosciences Bridgemapper program, we identified junction reads compatible with a tandem duplication mediated by these ribosomal operons. We infer that a duplication of this 820 kb region (including the ICR) occurred in the recipient strain prior to the recombination event with donor DNA. The *mrr*::*tetRA* transferred from the donor was then incorporated into one copy of the duplication, leaving the other copy of the duplication with the recipient ICR.

#### Genetic identification of ICR-only replacements

**Fig 6:**
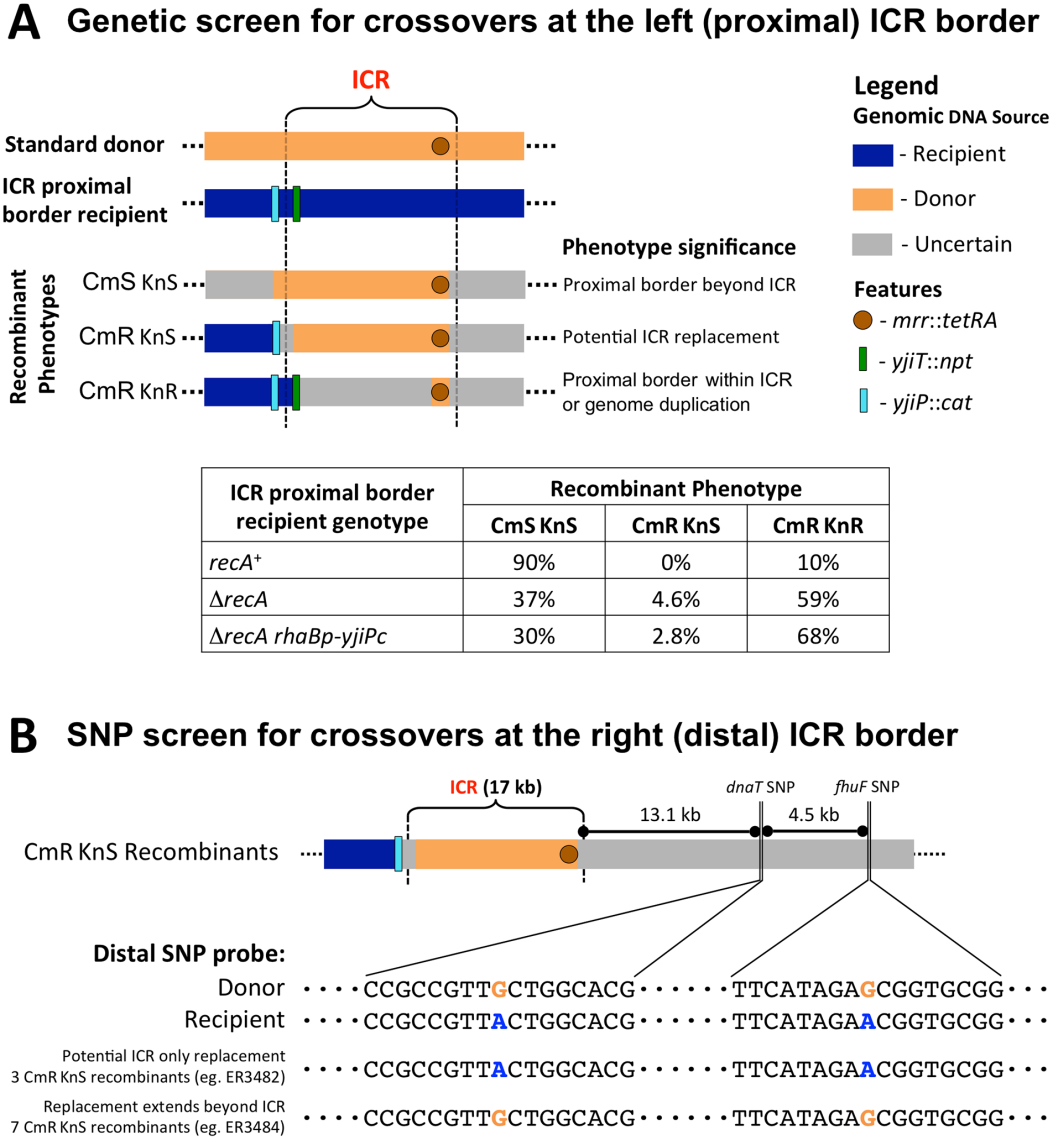
Identification of recombinants that replace the ICR only. (A) Schematic of the mating configuration designed to allow rapid genetic screening of the proximal ICR border status. In the recipient, A KnR cassette replaced a gene inside the proximal border of the ICR (*yjiT*::*npt*) and a CmR cassette replaced a nearby gene outside the proximal border (*yjiP*::*cat*). We call these ICR proximal border recipients. With this arrangement, a recombinant with donor DNA confined to the ICR will lose KnR but keep CmR and thus can be quickly identified by antibiotic screening. The embedded table displays the distribution of recombinant phenotypes in matings with different recipient backgrounds. The Δ*recA* ΔrepE* donor was crossed with recA^+^, Δ*recA* or Δ*recA yjiPc-rhaBp* proximal border recipients (Crosses 7, 8, and 10 respectively); at least 100 recombinants from at least three independent matings of each cross were tested. The proximal crossover yielding complete donor replacement (top configuration) dominated Rec+, with some recombinants showing proximal crossover within the ICR (bottom configuration). In *recA*, a larger fraction showed proximal crossover within the ICR, and a minority showed proximal crossover within the ICR border region (middle configuration). When YjiPc was overexpressed this distribution did not change greatly. (B) Detecting distal crossover events with PCR and Sanger sequencing. The schematic shows SNP configurations at the distal ICR border. Sanger sequencing of the closest two SNPs distal to the ICR in the ten KnS, CmR recombinants from Cross 8 showed that seven shared the SNPs of the donor, while three (ER3482, ER3483, and ER3510) shared the SNPs of the recipient. For these three, the distal-side limit of the replacement event lies within a ~13 Kb region that includes the downstream edge of the ICR.

We were particularly interested in detecting recombinants in which only the ICR was exchanged. To estimate the frequency of such limited replacement events, we designed a new recipient marker configuration that could facilitate the search for them. These recipients (ER3472, Rec^+^ and ER3473, Rec^-^) carry two antibiotic resistance cassettes flanking the proximal edge of the ICR, with the wild-type sequence of the rest of the ICR. Specifically, a kanamycin resistance cassette replaced *yjiT* within the ICR and a chloramphenicol resistance cassette replaced *yjiP* outside of it (Fig 2). Treating these cassettes as unselected markers allows us to screen TcR SmR recombinants for those with the CmR KnS phenotype (Fig 6A), which will only arise from a recombination event in which one edge of the replacement event is located within the 2.5 kb stretch of DNA that contains the proximal edge of the ICR, between *yjiP* and *yjiT*. Since the purpose of these recipients is to focus on the proximal border of the ICR, we referred to them as ICR proximal border genetic probe recipients. We mated the *recA* ^+^ and Δ*recA* recipients with this configuration with the Δ*recA* ΔrepE* donor (Crosses 7 & 8, respectively). Over 100 recombinants from each mating were screened for kanamycin and chloramphenicol sensitivity. The possible recombinant configurations and frequencies of these among the recombinants are shown in Fig 6A.

The control *recA* ^+^ cross (Cross 7) primarily produced recombinants that were sensitive to both drugs, suggesting that most recombination events replace a >20 kb segment of DNA that extends at least from *yjiP* to *mrr*. This agrees with the literature; recombinants from a Rec^+^ X Rec^+^ Hfr cross selecting for a marker within 500 kb of the *oriT* usually inherit donor DNA in a single large segment [31]. None of the recombinants from the *rec* ^+^ cross exhibited the KnS CmR phenotype resulting from a proximal crossover within the ~2.5 kb separating the two resistance cassettes. We infer that specific ICR replacement events are too rare to observe in a system that allows RecA-mediated recombination.

In the Δ*recA* cross (Cross 8), the phenotypic ratios were quite different. Only 37% of the events replaced both recipient antibiotic markers (KnS CmS) (Fig 6A). These resemble the *recA*-independent large patch replacement events we previously characterized via genomic sequencing analysis (Fig 5). Most recombinants (59%) retained both recipient markers (KnR CmR). Three mechanisms could play a role in generating these. A randomly-placed proximal crossover of the same kind seen in Fig 5 might occur within the 16 kb between *yjiT* and *mrr*; a site-specific activity might replace a subset of the region; or a genome duplication event in the recipient population could be followed by replacement of the region in one copy, as was observed in recombinant ER3455 (S7 Fig). The fraction of presumptive duplication recombinants in the mating that produced ER3455 (Cross 5, see Fig 3B) is low (9%) compared with the present result (59%), arguing against a role for duplications in the recipient. We lack informative sites that would distinguish between the site-specific and random possibilities.

Ten recombinants out of the 217 screened (4.6%) display proximal crossovers yielding the CmR KnS phenotype compatible with specific ICR replacement. To determine the position of the distal crossover events in these recombinants, we used PCR interrogation of the two SNPs that distinguish the recipient from the donor at 13 and 18 kb distal to the ICR (Fig 6B). In seven of the recombinants, these two SNPs matched that of the donor suggesting that the integrated donor DNA extends beyond the distal side of the ICR in these recombinants. The other three recombinants (which were each obtained from independent matings) matched the SNPs of the recipient. Thus, three of 217 recombinants result from crossovers in both ICR border intervals (2.5 kb proximal and 13.1 kb distal).

These three recombinants (ER3482, ER3483, and ER3510) are compatible with occurrence of targeted recombination event at the ICR. They may represent an enriched class. If crossovers occurred randomly throughout the genome, a recombinant exhibiting both crossovers within the ICR border intervals is 1 in 6600 or 0.015% (See S3 File for an explanation). Our matings produced recombinants with this crossover pattern at a 100-fold higher frequency (3/217 or 1.4%). This is a frequency of 3 X 10^-12^ CFU/recipient per hour. At present, the mechanism of such enrichment is unclear.

We further investigated how YjiPc might influence the types of *recA*-independent recombination events by repeating our ICR proximal border genetic probe mating experiments with *yjiPc* induction. A Δ*recA yjiP*::*cat yjiT*::*kan rhaBp-yjiPc recipient* was mated with the standard Δ*recA* ΔrepE* donor in 0.2% rhamnose, and over 100 recombinants were screened for kanamycin and chloramphenicol sensitivity (Cross 10; Fig 6B). Of the recombinants tested, 30% were CmS KnS, 68% were CmR KnR, and 2.8% were CmR KnS. These phenotypic frequencies are quite similar to those of recombinants from the mating without *yjiPc* induction (37%, 58%, and 4.6% respectively in Cross 8). Thus, we conclude that although *yjiPc* is a promoter of *recA*-independent recombination, there is no evidence that YjiP mediates targeting of the replacement events by itself.

## Discussion

In the present study, we developed a conjugal system to characterize mechanisms of horizontal gene transfer (HGT) starting with the model organism *E*. *coli* K-12. We discovered a novel low-frequency event class and implicated an uncharacterized protein family in promoting this class. We also identified genetic elements that may promote RecA-independent recombination.

### Large RecA-independent genomic replacements are a novel recombinant class

Large RecA-independent genomic replacements (up to 2.4 Mb of the recipient chromosome) have not been reported previously. A recent paper explains how to use large genomic replacements to create chimeric genomes [46], but the study is in a *recA* ^+^ background. Other research groups study RecA-independent recombination, but focus on the replacement of a selective marker or at specific sites [47,48]. These are infrequent, and their detection here was dependent on whole-genome sequence assembly with the SMRT platform, a technology only recently available at reasonable cost.

To characterize the mechanism by which large replacements occur we combine our findings with the literature to draw some conclusions. Our first step in understanding this mechanism is to categorize this recombination event into one of the three classical recombination models: Break-join (homologous strand exchange), copy-choice (template exchange occurs during replication), or break-copy (a hybrid of the two) [49]. Crossovers in the six sequenced recombinants exhibiting large genomic replacements were all localized to regions of homology. Furthermore, sequencing did not reveal any “novel joints” in which donor DNA is connected to recipient DNA at a new point [50]. These observations support a copy choice model of homology-directed recombination, but do not refute the other models.

Several RecA-independent homology directed recombination mechanisms that follow the copy-choice model have been proposed by Lovett and coworkers [51]. These mechanisms involve a replication “slippage” event in which the polymerase temporarily dissociates from its original template allowing the nascent strand to anneal to a different homologous template before replication resumes. For our events, a DNA polymerase could switch homologs, for example on encountering a template nick. Resolution by for a distal crossover could be carried out in the same way, or another kind of resolution could reunite the replication complex with the recipient chromosome. In the context of this model, YjiP could promote recombination in several ways: by facilitating resolution of crossed strands (a structure specific nuclease activity), by nicking a template strand, or by exonuclease action at a broken fork to reveal a strand for annealing.

### YjiP represents a novel class of DNA modifying enzymes

This study is the first experimental report to illuminate the role of YjiP. The small amount of bioinformatic evidence available is compatible with our demonstration that it can enhance RecA-independent recombination. It was classified as a putative transposase (Transposase_31) by the TigrFam database because of its distribution and conservation [52]. A family of related proteins is included in the Pfam database (PF04754) [53], where it is classified within a clan (PDDEXK domain) containing many phosphodiesterases [54].

YjiP homologs are prevalent in Enterobacteriaceae (*E*. *coli*, *Salmonella*, *Yersinia*, *Shigella*, ect.) and occur sporadically among other taxa [53,55]. Strains that have a copy of *yjiP* often contain multiple paralogs, which tend to be more similar to each other than to homologs in other species [52]. *E*. *coli* K-12 encodes 5 paralogs (YjiP, YadD, YfcI, YfaD, and YhgA); the *yadD* gene appears to have inserted into the *panBCD* operon relatively recently [56]. This distribution pattern is similar to that of mobile elements and their associated genes. Expression experiments in *Escherichia coli* also suggest a connection between *yjiP* and biofilms and quorum sensing [57]. A *yjiP* null strain exhibits ~50% reduction in biofilm formation [58] and *yjiP* is upregulated during the initial stages of biofilm construction. This connection is relevant to the biology of YjiP’s role in recombination because DNA transfer (a precondition for HGT) is enhanced in biofilms [5].

The putative phosphodiesterase activity of YjiPc provides a potential explanation for reduced viability seen in our experiments (Fig 4C & S4E Fig). An overexpressed nuclease could cause DNA damage, especially toxic in a cell unable to repair DNA via the efficient RecA-dependent pathways [59]. The observation that *yjiPc* overexpression does not reduce viability in a *recA* ^+^ background but does induce an SOS reporter supports this hypothesis.

We conclude that YjiP is part of an experimentally uncharacterized class of DNA modifying enzymes that contribute to HGT, consistent with the bioinformatic context described above. Its mechanism of action is unclear, but likely includes nuclease activity. YjiP’s function does not seem to be targeted to the ICR under our conditions. Notably, both F' formation and novel large replacements are stimulated by its expression. Other ICR framework genes (*yjiRS* and *yjiAXY*) might contribute targeting activity, resulting in SSR at this locus. Future studies will also focus on the YjiP paralogs in *E*. *coli* and how they might influence both recombination and cell viability.

### Genetic elements that could affect recombination found in crossover intervals

A distinguishing aspect of this study is the inventory of 447 informative variant sequence positions. This allowed us to define proximal and distal crossover intervals where DNA exchange occurred in 6 recombinants (3 WT and 3 *yjiPc* induced). Several features found within these 12 crossover intervals deserve further discussion.

The sequence interval between the initial DNA entry (*yhjP*) and the selected marker (*mrr*::*tetAR*) defines the region in which the first (proximal) crossover is most likely to occur. There are six variant positions in this stretch, three containing sites of action for DNA transactions and coding sequences for the corresponding proteins. One of these elements is the invertible segment *fimS* together with the FimE and FimB recombinases that act on it, encoded in flanking genes [43,44]. The other two are the IS1 and IS5 elements encoding RecA-independent transposases (InsAB and InsH, respectively), and containing their sites of action. FimE, FimB, IS1, and IS5 all have the capacity to induce DNA breaks and ligate them at specific loci within the proximal crossover intervals. Normal action would result in inversion, transposition or adjacent deletion formation mediated by the mobile element. During conjugation, a large segment of homologous DNA is introduced; possibly such activity could contribute to the formation of an intermediate complex such that it connects recipient DNA to donor DNA, then to be acted on by YjiP or one of the four paralogs in K-12. In support of this theory is the fact that recombinant ER3454 has a novel *fimS* sequence compared to both the donor and the recipient, suggesting that the proximal crossover of this recombinant occurred within *fimS*. Such interactions have not been described previously, but since the events studied occur at very low frequencies, novel possibilities may be entertained.

For the second (distal) crossovers required to recover viable progeny, 50% of them occurred in the same 44.5 kb interval (S3 Table) suggesting the presence of a recombination hotspot. The most notable element found within this region was *dif*, located in the replication terminus [60]. This is the site of action of the site-specific XerCD recombinase. Xer-dif recombination decatenates chromosome copies during cell division [61][62]. This activity would therefore abridge any recombinants with distal crossovers past the dif site, by connecting the recipient homolog to the donor thus resolving a forked chromosome created by the proximal crossover that initiated the recombination event.

### Relevance to evolutionary processes

This novel type of recombination could be significant factor in genomic evolution. The recent surge in bioinformatic studies has begun to show that natural chromosome transfer is much more prevalent than previously believed [63]. Novel Integrative conjugal elements that transfer chromosomal DNA are rapidly being discovered in a variety of bacteria ranging from *Mycoplasma* [64] *to Streptococcus* [65], to *Yersinia* [66]. Some of these systems are even less constrained and more efficient than Hfr conjugation from *E*. *coli* [66]. Although the RecA-independent events that we characterized are rare, the abundance of conjugation in nature gives these events ample opportunity to modify genomes. The events described here have also not shown a requirement for extensive homology and could occur between distantly related strains where homologous recombination would not be effective.

### System qualification

Using different configurations of the conjugal system, we estimated relative event frequencies for known recombination events that we could identify (Table 2). These include homologous recombination, F’-plasmid formation, and large RecA-independent genomic replacement. Genome duplications mediated by ribosomal operons were also recovered as background events. We found that replacement of a gene island of particular interest (immigration control region (ICR)), likely occurs, but most of the recombination events we observed were not specific to the ICR.

**Table 2:** Frequencies of recombination events in the conjugal system.

Recombination Event (frequency)	Recombination Subcategory	% of total Recombinants	*yjiP* induction increases frequency?
Homologous Recombination (0.21 CFU/recipient per hour)	Recombination beyond ICR borders	90%	No
	Recombination within ICR or into genomic duplication	10%	No
	Site specific ICR replacement	0.0%	NA
F'-plasmid formation (3.5 X 10^-6^ CFU/recipient per hour)	Recipient ICR replaced	9.6%	Yes
	Recipient ICR maintained	90%	Yes
RecA-independent genomic exchange (2.1 X 10^-10^ CFU/recipient per hour)	Recombination beyond ICR borders	40%	Yes
	Recombination within ICR	50%	Yes
	Genome duplication before recombination	8.5%	Yes
	Site specific ICR replacement	1.4%	Yes

### The conjugal system is an effective genetic tool for analyzing HGT

The conjugal system we developed has proven to be an extremely versatile tool for studying HGT. It is robust to the point where rare recombination events can be isolated and has the flexibility to focus on specific genetic elements, gene islands, or proteins of interest. It can also be expanded to analyze HGT between more distantly related strains where homology-based recombination is much less of a factor. We hope that other labs studying genomic evolution, DNA recombination, or other related fields will recognize the utility of our system and begin to use it for their own research purposes.

## Supporting Information

S1 Fig Gene content of the Immigration control region (ICR) in three strains of *E*. *coli* and in *Salmonella enterica sv typhimurium* LT2. The content of the ICR region tends to be highly variable, even among closely related strains. Boxes of the same color in different strains represent orthologous genes. Genes were judged orthologous if nucleotide similarity was >65%. Type IA and Type IB restriction systems are paralogous. This variability is in sharp contrast to the conserved framework genes (*yjiPRS* & *yjiAXY*) suggesting that a site-specific mechanism could be involved in exchange of all or part of the ICR. Image adapted from [22].
(TIF)

S2 Fig Recovery of F' plasmids from crosses with *repE* ^*+*^donors: Short read sequence overrepresentation. Four recombinant genomes from the Δ*recA* donor X *recA13* recipient mating (Cross 4) were sequenced with Illumina and aligned to an *in silico* model of the donor (ER3276) genomic sequence. The relative coverage of (A) the entire genome and (B) the genomic region surrounding the F factor and ICR loci is shown. Disagreement between the donor reads and the *in silico* model revealed the presence of a deletion ((Δ*ori1* = Δ*(pifA-yddA*)) covering one of the two vegetative origins of the F plasmid. All recombinants exhibit an approximate 2-3X increase of coverage, from the start of the proximal F region to a variable point beyond the ICR. We infer the presence of an F’ plasmid carrying the overrepresented region. Each F’-plasmid is distinct in the extent of genomic DNA that it carries. See S2 File for discussion.
(TIF)

S3 Fig Deleting the leading F-DNA prevents formation of F’-plasmids. (A) DNA transferred from *oriT* as far as the selected ICR by the original Δ*recA* donor (ER3276) and by the donor in which the leading F-DNA has been deleted (ER3435). The leading F DNA deletion is denoted ΔrepE*; noted that the deletion removes 49 genes including the toxin-antitoxin systems *ccdAB* and *flmAB*. (B) Depiction of the recombinants derived from each donor. Genetic and sequence analysis revealed an F’-plasmid with a stable copy of *tetRA* in all recombinants created by the original donor. The chromosomal ICR typically kept its *npt* cassette, but a small proportion of recombinants replaced it with a *tetRA* cassette. In recombinants created with the ΔrepE* donor, the F’-plasmid did not form and the *yjiT*-*mrr*::*npt* marker on the recipient genome was usually replaced with the *mrr*::*tetRA* marker from the donor.
(TIF)

S4 Fig Further analyzing the effect of *yjiP* overexpression on cell viability and mating recombination efficiency. (A) Expression dynamics of a surrogate reporter. The β-galactosidase activity of a *rhaBp-lacZ* construct in *E*. *coli* (ER3340) after induction with rhamnose. Cultures were grown at 37°C with shaking and treated with 0.2% rhamnose at an OD_600_ of 0.2. β-galactosidase assays of culture samples were then taken at regular intervals over the next 6.5 hours. The addition of rhamnose increased the accumulation of β-galactosidase ~200 fold compared to an untreated control and took about 225 min to reach full expression. (B) The frequency of recombination during matings between the Δ*recA* ΔrepE* donor and *a rec* ^*+*^ *rhaBp-yjiPc* recipient (cross 9) with and without 0.2% rhamnose. Recombination efficiency was calculated as the frequency of recombinant formation per viable recipient per hour in the mating mixture. Inducing *yjiP* expression with rhamnose did not significantly affect recombination efficiency in a *rec* ^+^ background. (C) The change in recombination efficiency due to rhamnose treatment in matings between the Δ*recA repE* ^+^ donor and a *recA13* recipient (Cross 4) or between that donor and *recA13* recipients with with rhamnose inducible copies of either *yjiA* (cross 11; *rhaBp-yjiA*) or *yjiPc* (cross 12; *rhaBp-yjiPc)*. As with the other matings, inducing *yjiPc* expression increased recombination efficiency around 4 fold, but rhamnose had no effect on the control recipient or the *yjiA* inducible recipient. (D) Cell growth of a *recA*13 recipient containing the *rhaBp-yjiPc* construct (ER3336) treated with and without 0.2% rhamnose as measured by OD_600_ readings. Although *yjiPc* induction reduces the ability of recipients to form colonies on selective media (Fig 4B–4C), OD_600_ readings remain unaffected by rhamnose treatment. (E) *yjiP* overexpression induces an SOS response in *E*. *coli*. A *rec* ^+^ strain carrying both the *rhaBp-yjiPc* construct and a *dinDp-lacZ* reporter of the SOS response (ER3544; [39]) on X-gal media with and without 0.2% rhamnose. Colonies were substantially more blue in the presence of rhamnose than in its absence.
(TIF)

S5 Fig Variant maps allow parent of origin assignment in recombinants. The donor and recipient genomes display about 1 variation per 10 kb (box 1). When a recombinant genome (ER3445) is aligned to the recipient genome using Mauve, variations are observed where donor genomic DNA has been incorporated (box 2). The inverse pattern is seen when the recombinant is compared with the donor (box 3). This display allows assignment of DNA stretches to each parent (box 4). Since variations are separated by a ~10000 bps this analysis still leaves a small region of DNA of uncertain origin (grey coloring). Presumably, the DNA crossover events occur in these uncertain intervals, designated "crossover intervals".
(TIF)

S6 Fig Variations between the donor (ER3435), recipient (ER3440/ER3460), and recombinants (ER3445, ER3446, ER3454, ER3466, ER3475, and ER3476) from the distal *yjhP* SNP to the *yjiC*/IS5 variation. Variations that match the donor genome are orange and those matching the recipient genome are blue. The *fimS* invertible segment, labelled *fimInv* here, is present in opposite orientations in donor and recipient. The gray FimInv* element in recombinant ER3454 is a unique sequence compared to both the donor and the recipient. This analysis was performed with the Geneious R7 software and was used to determine the proximal crossover intervals described in S3 Table.
(TIF)

S7 Fig Coverage of reads from Pacific Biosciences sequencing of the KnR recombinant ER3455 produced by Cross 5. Sequences were assembled using the RS_Bridgemapper.1 algorithm with either the (A) Δ*recA* ΔrepE* donor (ER3435) or (B) Δ*recA* (ER3440) recipient genomes as the reference sequence. An area of 2X coverage between the *rrlE* and *rrsH* ribosomal subunit encoding genes indicates that a duplication of this region is present. When compared with the donor, the ICR drops to single copy levels. This recombinant contains no F DNA, and has lost the Rac prophage. We infer that the duplication occurred in the recipient strain prior to the recombination event with donor DNA. The duplicated region then integrated the *mrr*::*tetRA* transferred from the donor into one copy of the recipient’s ICR, leaving the other ICR with the *yjiT-mrr*::*npt* construct unaffected.
(TIF)

S1 Table Strains, plasmids, and oligonucleotides used in this study. (DOCX)

S2 Table Mating partners and conditions used in this study. (DOCX)

S3 Table Genes and relevant features within crossover intervals of the large patch RecA-independent genomic replacement recombinants. (DOCX)

S1 File Supplemental methods. (DOCX)

S2 File Validation of the conjugal system. (DOCX)

S3 File Calculating the expected frequency of ICR-specific replacement. (DOCX)
