# Correction: Novel *recA*-Independent Horizontal Gene Transfer in *Escherichia coli* K-12

**DOI:** 10.1371/journal.pone.0143991

**Published:** 2015-11-30

**Authors:** Anthony W. Kingston, Chloé Roussel-Rossin, Claire Dupont, Elisabeth A. Raleigh

There is an error in Fig 4A. Please see the corrected Fig 4 here.

**Fig 4:**
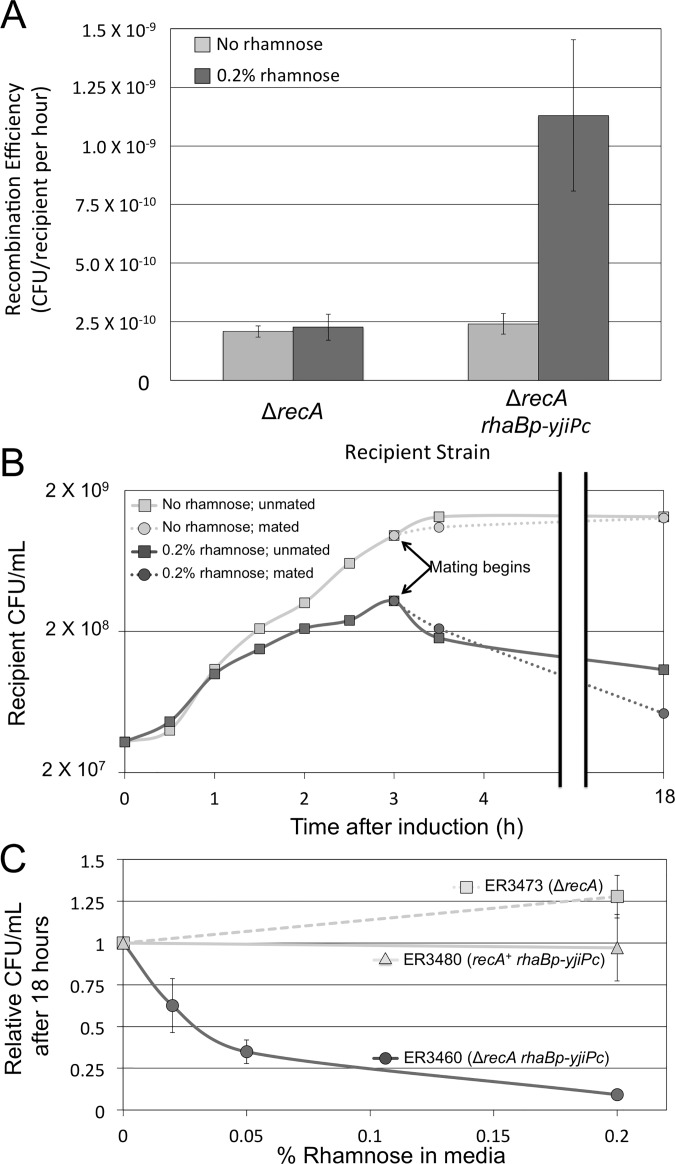
YjiP promotes *recA*-independent recombination and reduces cell viability. (A) The frequency of recombination during matings between the ΔrepE* Δ*recA* donor and either the Δ*recA* recipient (cross 6; Δ*recA*) or a Δ*recA* recipient with inducible overexpression of *yjiP* (cross 13; Δ*recA rhaBp-yjiPc*), with and without 0.2% rhamnose. Recombination efficiency was calculated as the frequency of recombinant formation per viable recipient per hour in the mating mixture. Inducing *yjiPc* expression with rhamnose significantly increases recombination efficiency ~5 fold (P-value = 0.018), but rhamnose has no significant effect when the recipient lacks the *rhaBp-yjiPc* gene fusion. (B) Cell viability over time of the Δ*recA rhaBp-yjiPc*recipient (ER3460) grown in 0.2% rhamnose. This experiment was performed three times with biological replicates, but only the results of a single representative trial are shown for clarity. Rhamnose was added at t = 0, and cells were mated after 3 hours of growth with ER3435. Untreated and unmated cells were also included as controls. Rhamnose-induced*yjiPc* expression reduces cell proliferation for the first three hours after treatment and begins to kill cells afterwards. Mating did not significantly affect cell viability. (C) Dose-response of cell killing: fraction starting titer for three strains at 18 hours as a function of inducer concentration. Strains were Δ*recA* (ER3473), *recA* ^+^ *rhaBp-yjiPc* (ER3480), and Δ*recA rhaBp-yjiPc* (ER3460) grown in various concentrations of rhamnose for 18 hours relative to an untreated control. Higher concentrations of rhamnose are increasingly lethal to ER3460; 0.2% rhamnose kills ~90% of the normally viable cells. All experiments in panels A and C were performed with a minimum of three biological replicates with error bars representing standard error.
