# Supplementary figures and images for: Novel recA-Independent Horizontal Gene Transfer in Escherichia coli K-12
Source: PLoS One. 2015 Jul 10;10(7):e0130813. doi: 10.1371/journal.pone.0130813 (PMC4498929; doi:10.1371/journal.pone.0130813)

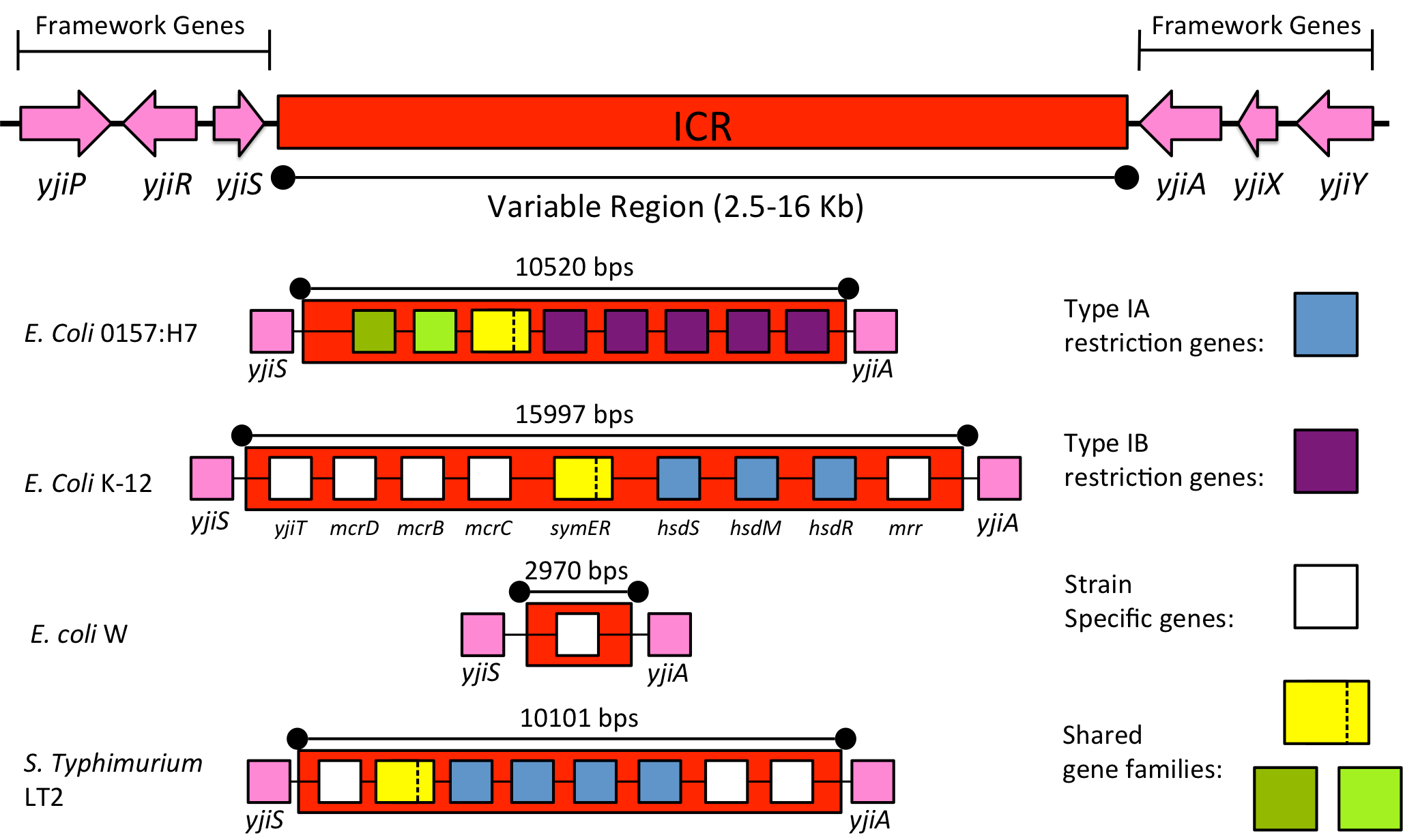

Supplement: S1 Fig — The content of the ICR region tends to be highly variable, even among closely related strains. Boxes of the same color in different strains represent orthologous genes. Genes were judged orthologous if nucleotide similarity was >65%. Type IA and Type IB restriction systems are paralogous. This variability is in sharp contrast to the conserved framework genes (yjiPRS & yjiAXY) suggesting that a site-specific mechanism could be involved in exchange of all or part of the ICR. Image adapted from [22]. (TIF) [file pone.0130813.s001.tif]

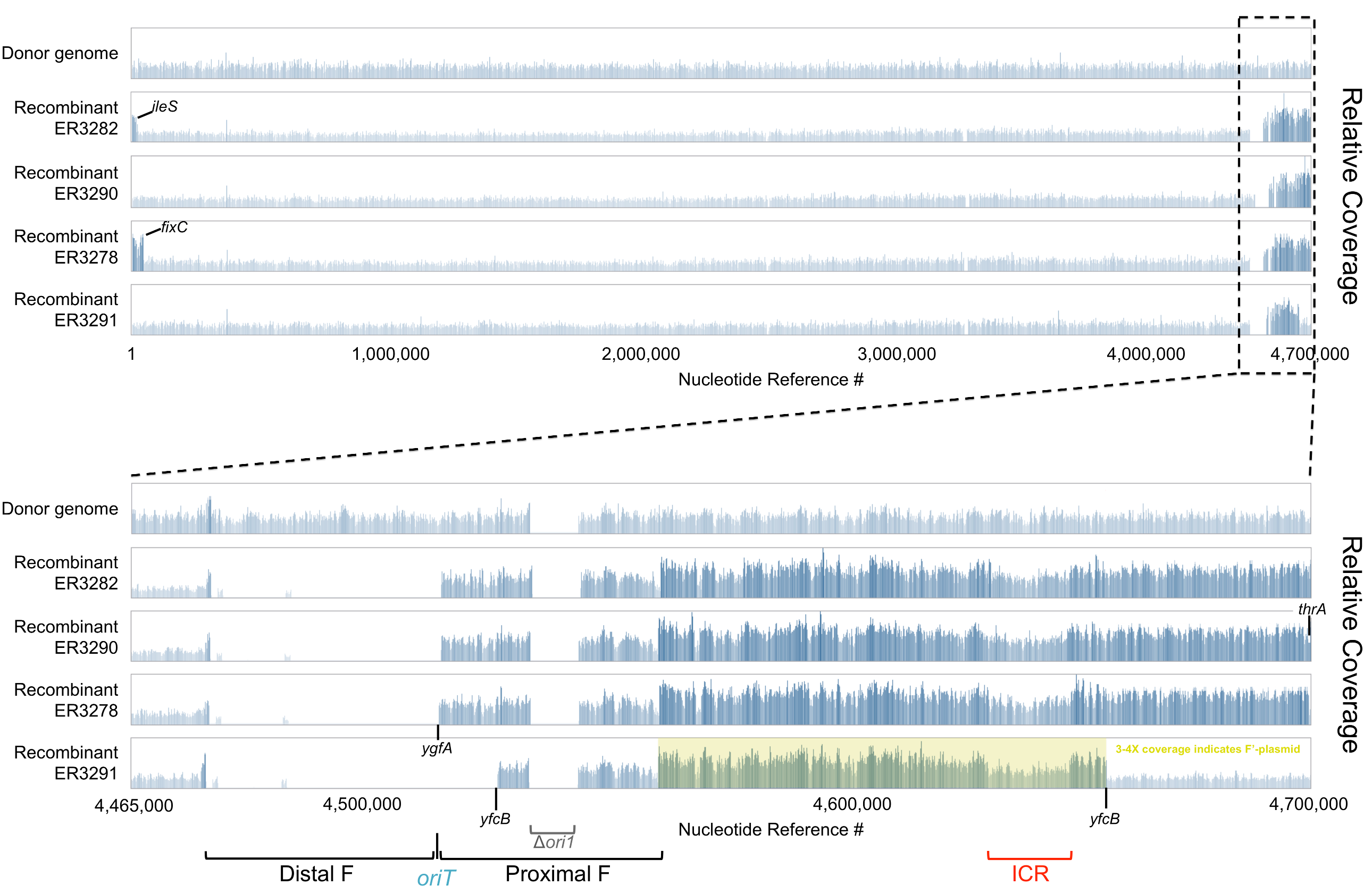

Supplement: S2 Fig — Four recombinant genomes from the ΔrecA donor X recA13 recipient mating (Cross 4) were sequenced with Illumina and aligned to an in silico model of the donor (ER3276) genomic sequence. The relative coverage of (A) the entire genome and (B) the genomic region surrounding the F factor and ICR loci is shown. Disagreement between the donor reads and the in silico model revealed the presence of a deletion ((Δori1 = Δ(pifA-yddA)) covering one of the two vegetative origins of the F plasmid. All recombinants exhibit an approximate 2-3X increase of coverage, from the start of the proximal F region to a variable point beyond the ICR. We infer the presence of an F’ plasmid carrying the overrepresented region. Each F’-plasmid is distinct in the extent of genomic DNA that it carries. See S2 File for discussion. (TIF) [file pone.0130813.s002.tif]

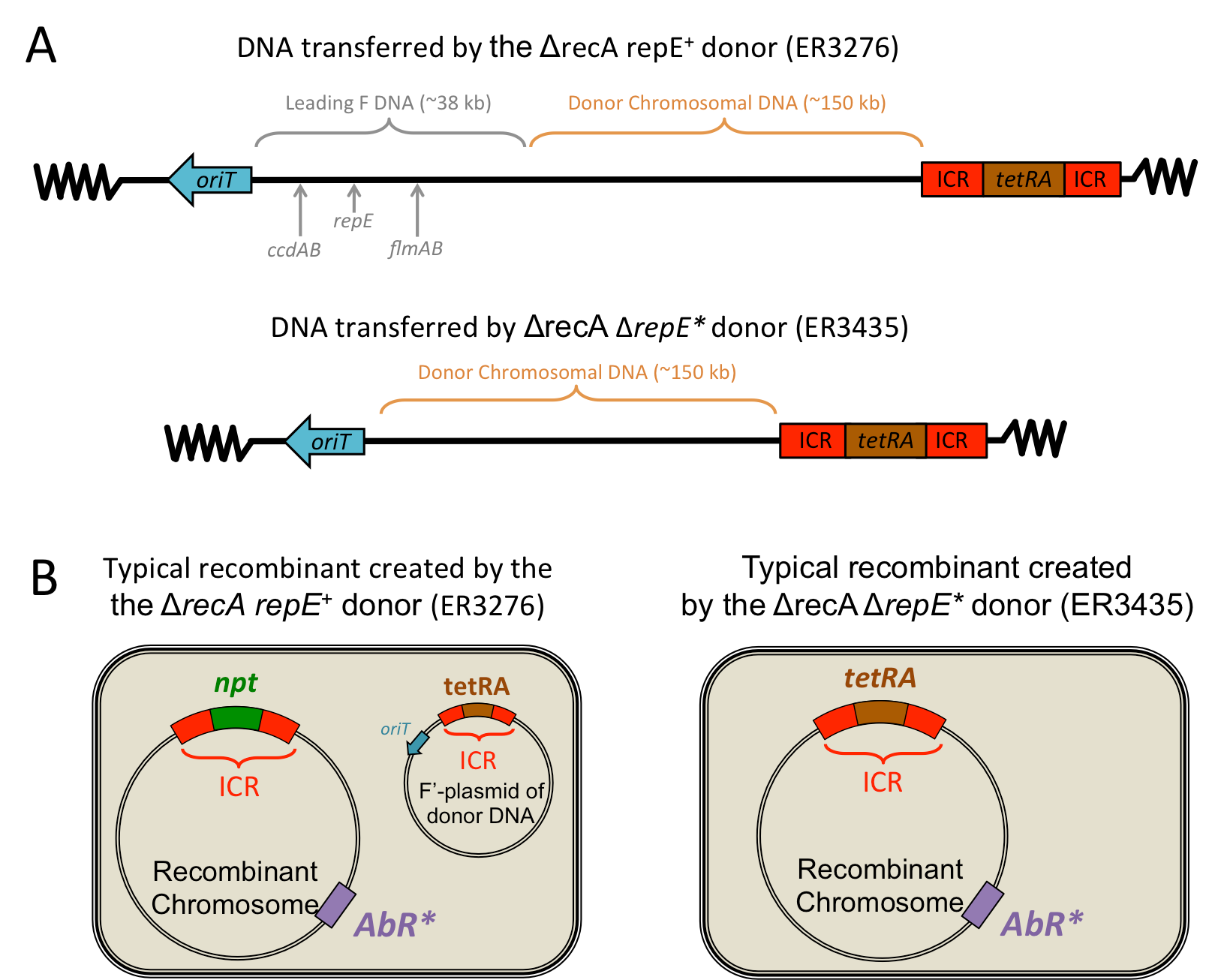

Supplement: S3 Fig — (A) DNA transferred from oriT as far as the selected ICR by the original ΔrecA donor (ER3276) and by the donor in which the leading F-DNA has been deleted (ER3435). The leading F DNA deletion is denoted ΔrepE*; noted that the deletion removes 49 genes including the toxin-antitoxin systems ccdAB and flmAB. (B) Depiction of the recombinants derived from each donor. Genetic and sequence analysis revealed an F’-plasmid with a stable copy of tetRA in all recombinants created by the original donor. The chromosomal ICR typically kept its npt cassette, but a small proportion of recombinants replaced it with a tetRA cassette. In recombinants created with the ΔrepE* donor, the F’-plasmid did not form and the yjiT-mrr::npt marker on the recipient genome was usually replaced with the mrr::tetRA marker from the donor. (TIF) [file pone.0130813.s003.tif]

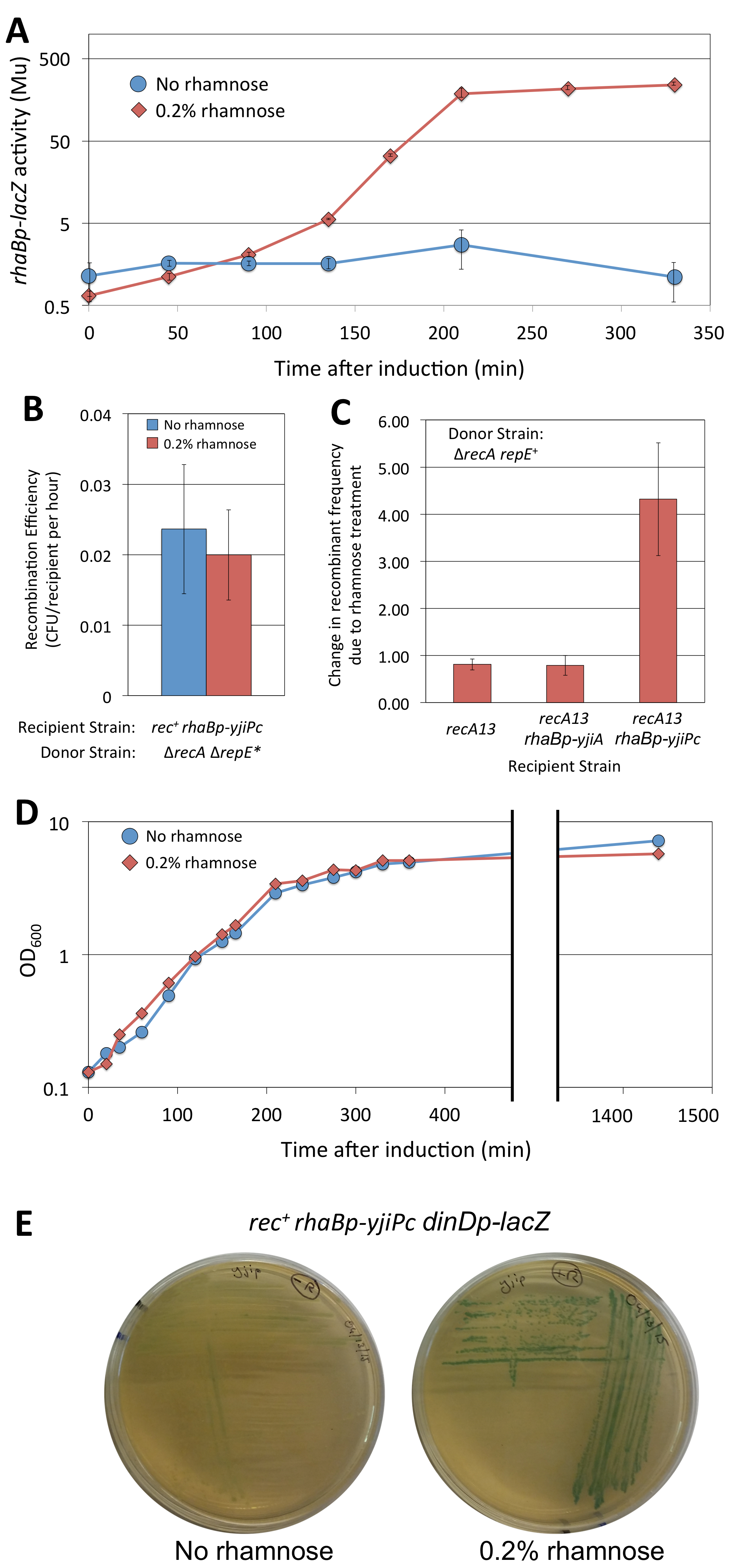

Supplement: S4 Fig — (A) Expression dynamics of a surrogate reporter. The β-galactosidase activity of a rhaBp-lacZ construct in E. coli (ER3340) after induction with rhamnose. Cultures were grown at 37°C with shaking and treated with 0.2% rhamnose at an OD600 of 0.2. β-galactosidase assays of culture samples were then taken at regular intervals over the next 6.5 hours. The addition of rhamnose increased the accumulation of β-galactosidase ~200 fold compared to an untreated control and took about 225 min to reach full expression. (B) The frequency of recombination during matings between the ΔrecA ΔrepE* donor and a rec + rhaBp-yjiPc recipient (cross 9) with and without 0.2% rhamnose. Recombination efficiency was calculated as the frequency of recombinant formation per viable recipient per hour in the mating mixture. Inducing yjiP expression with rhamnose did not significantly affect recombination efficiency in a rec + background. (C) The change in recombination efficiency due to rhamnose treatment in matings between the ΔrecA repE + donor and a recA13 recipient (Cross 4) or between that donor and recA13 recipients with with rhamnose inducible copies of either yjiA (cross 11; rhaBp-yjiA) or yjiPc (cross 12; rhaBp-yjiPc). As with the other matings, inducing yjiPc expression increased recombination efficiency around 4 fold, but rhamnose had no effect on the control recipient or the yjiA inducible recipient. (D) Cell growth of a recA13 recipient containing the rhaBp-yjiPc construct (ER3336) treated with and without 0.2% rhamnose as measured by OD600 readings. Although yjiPc induction reduces the ability of recipients to form colonies on selective media (Fig 4B–4C), OD600 readings remain unaffected by rhamnose treatment. (E) yjiP overexpression induces an SOS response in E. coli. A rec + strain carrying both the rhaBp-yjiPc construct and a dinDp-lacZ reporter of the SOS response (ER3544; [39]) on X-gal media with and without 0.2% rhamnose. Colonies were substantially more blue in the presence [file pone.0130813.s004.tif]

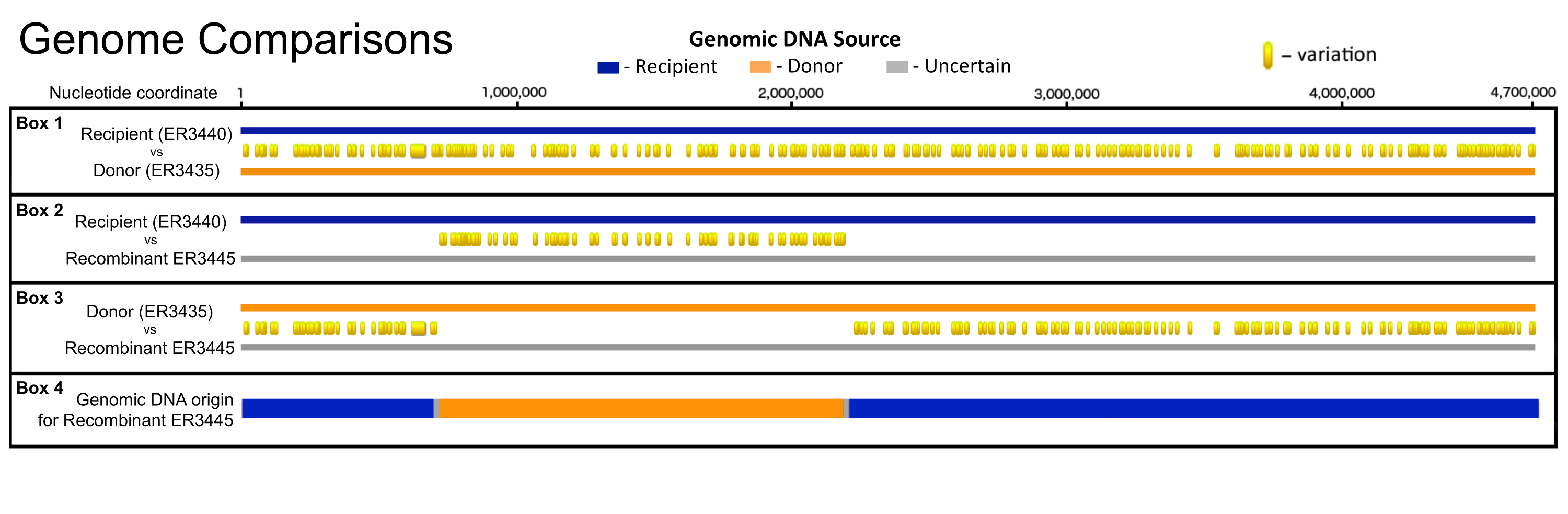

Supplement: S5 Fig — The donor and recipient genomes display about 1 variation per 10 kb (box 1). When a recombinant genome (ER3445) is aligned to the recipient genome using Mauve, variations are observed where donor genomic DNA has been incorporated (box 2). The inverse pattern is seen when the recombinant is compared with the donor (box 3). This display allows assignment of DNA stretches to each parent (box 4). Since variations are separated by a ~10000 bps this analysis still leaves a small region of DNA of uncertain origin (grey coloring). Presumably, the DNA crossover events occur in these uncertain intervals, designated "crossover intervals". (TIF) [file pone.0130813.s005.tif]

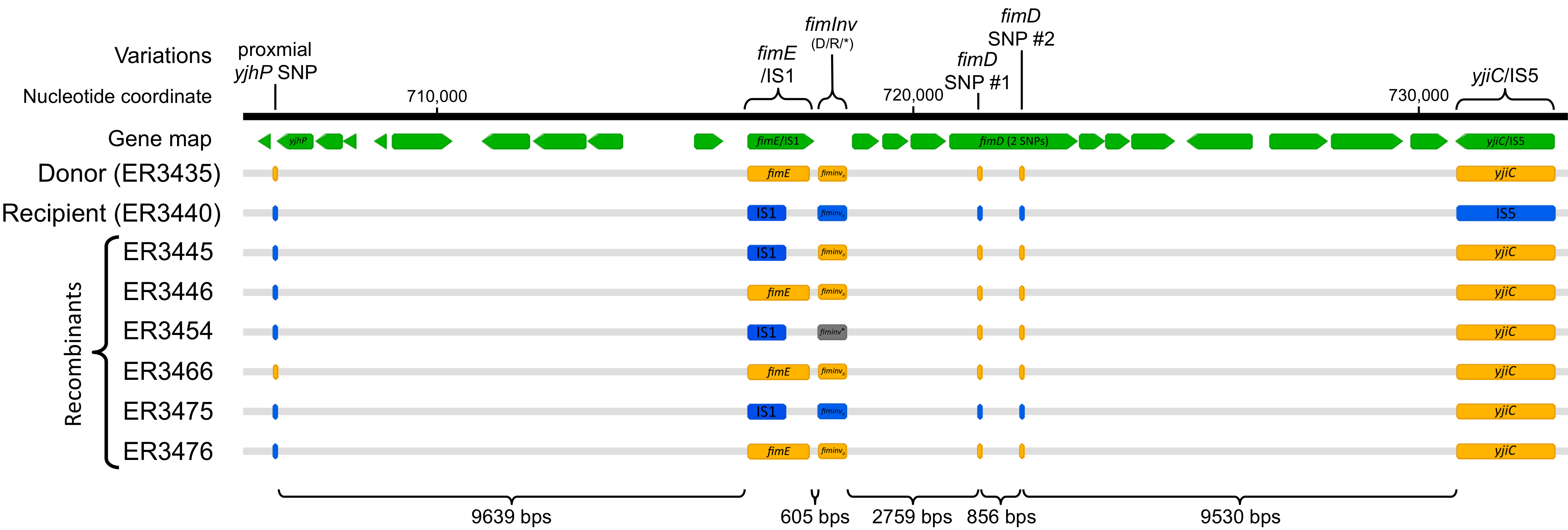

Supplement: S6 Fig — Variations that match the donor genome are orange and those matching the recipient genome are blue. The fimS invertible segment, labelled fimInv here, is present in opposite orientations in donor and recipient. The gray FimInv* element in recombinant ER3454 is a unique sequence compared to both the donor and the recipient. This analysis was performed with the Geneious R7 software and was used to determine the proximal crossover intervals described in S3 Table. (TIF) [file pone.0130813.s006.tif]

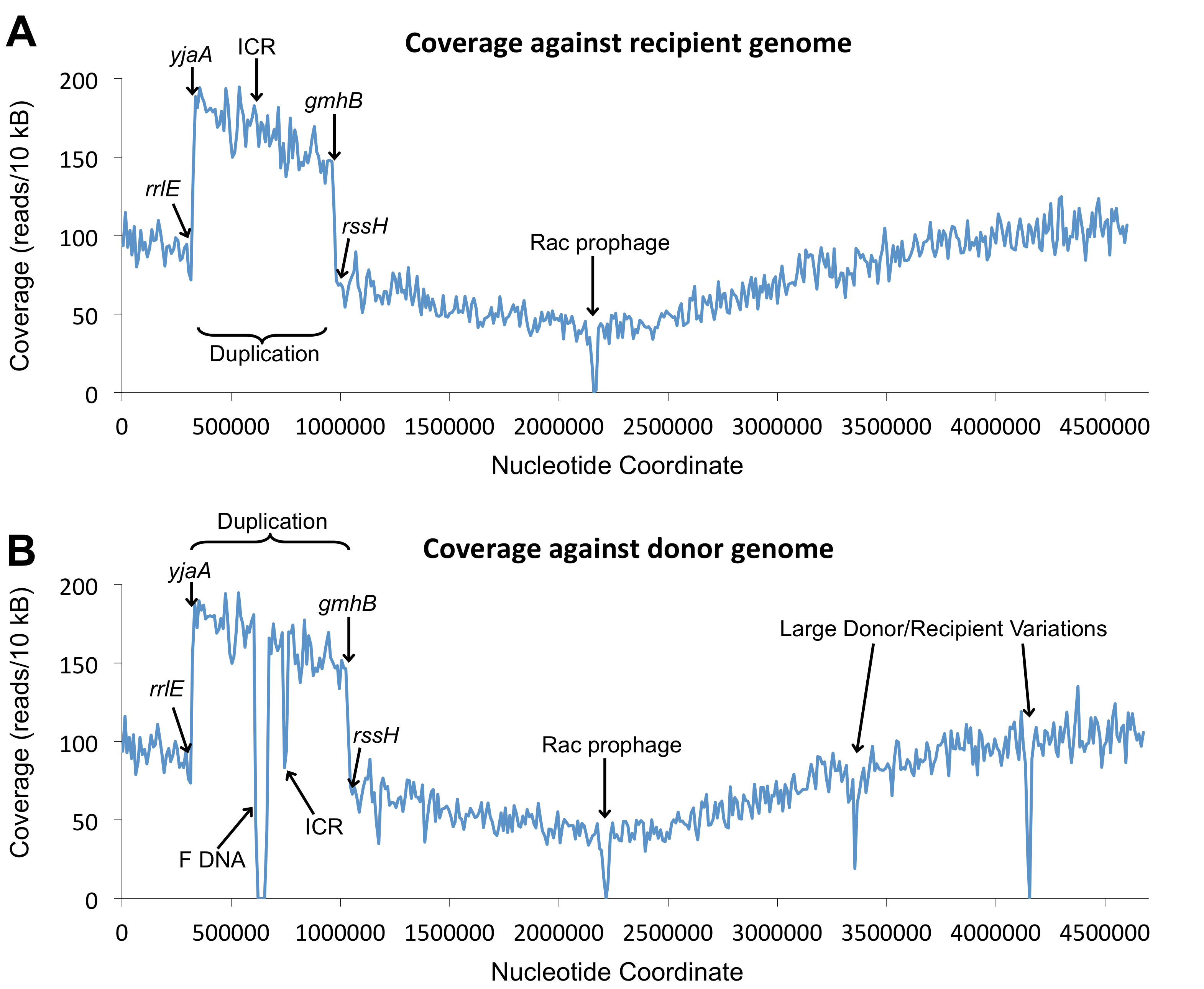

Supplement: S7 Fig — Sequences were assembled using the RS_Bridgemapper.1 algorithm with either the (A) ΔrecA ΔrepE* donor (ER3435) or (B) ΔrecA (ER3440) recipient genomes as the reference sequence. An area of 2X coverage between the rrlE and rrsH ribosomal subunit encoding genes indicates that a duplication of this region is present. When compared with the donor, the ICR drops to single copy levels. This recombinant contains no F DNA, and has lost the Rac prophage. We infer that the duplication occurred in the recipient strain prior to the recombination event with donor DNA. The duplicated region then integrated the mrr::tetRA transferred from the donor into one copy of the recipient’s ICR, leaving the other ICR with the yjiT-mrr::npt construct unaffected. (TIF) [file pone.0130813.s007.tif]
