# Supplementary material for: Novel recA-Independent Horizontal Gene Transfer in Escherichia coli K-12
Source: PLoS One. 2015 Jul 10;10(7):e0130813. doi: 10.1371/journal.pone.0130813 (PMC4498929; doi:10.1371/journal.pone.0130813)
Supplement: S1 Table — (DOCX) [file pone.0130813.s008.docx]

**S1 Table. Strains, plasmids, and oligonucleotides used in this study.**

| **Strain** | **Other name** | **F status** | **genotype^a^** | **plasmid** | **Source, reference, construction** |
| --- | --- | --- | --- | --- | --- |
| ER1370 |  | - | *fhuA2*::IS2 Δ(*lacZ*)*4826 glnX44 e14- trpE31* Δ(*hisG*)*1 argG6*(*FS*) *rpsL104*(*StrR*) *xyl-7 mtlA2*(*Fs*) *metB1*(*FS*) *serB28* |  | This lab [1] |
| ER1505 | CGSC4474; W3110 | - | *IN*(*rrnD-rrnE*)*1 rph-1* |  | CGSC [2-4] |
| ER1506 | CGSC6300; MG1655 | *-* | *rph-1* |  | CGSC [5] |
| ER1547 | V325 | - | *galk2 galT22 rpsL179 lac argA*::*Tn10 recD1014* |  | G.R. Smith [6] |
| ER1572 |  |  | *fhuA2*::IS2 Δ(*lacZ*)*4826 glnX44 trpE31* Δ(*hisG*)*1 rpsL104*(*StrR*) *xyl-7 mtlA2*(*Fs*) *metB1*(*FS*) *serB28* |  | ER1370 X P1*vir*(ER1505) → Arg+ |
| ER1607 | NM554 | - | Δ(*araA-leu*)*7697 [araD139]B/r* Δ(*codB-lacI*)*3 galK16 galE15*(*GalS*) *e14- mcrA0 relA1 recA13 rpsL150*(*StrR*) *spoT1 mcrB1 hsdR2* |  | N. Murray [7] |
| ER1629 |  | - | *fhuA2*::IS2 Δ(*lacZ*)*4826 glnX44 trpE31* Δ(*hisG*)*1 recD1014 argA*::*Tn10*(*TetR*) *rpsL104*(*StrR*) *xyl-7 mtlA2*(*Fs*) *metB1*(*FS*) *serB28* |  | ER1572 X P1*vir*(ER1547) → TcR (Arg- RecD-) |
| ER1636 |  | - | *fhuA2*::IS2 Δ(*lacZ*)*4826 glnX44 trpE31* Δ(*hisG*)*1 recD1014 rpsL104*(*StrR*) *xyl-7 mtlA2*(*Fs*) *metB1*(*FS*) *serB28* |  | ER1629 → revert Arg+ (TcS) |
| ER1954 | CGSC259, AB259, HfrH, HfrC | HfrPO1  (*∆(pifA-yddA*) | *supQ80 e14-* (*McrA-*) *relA1 spoT1 thiE1* |  | CGSC [1] |
| ER2170 |  | - | *∆(argF-lac)U169 glnX44 mcr-67 rfbD1? relA1? endA1 spoT1? dinD2::MudI1734 (KanR, lacZ(ts)) thi-1 ∆([fimB or yjiT]-opgB)114::IS10* |  | [8] |
| ER2204 | DH5aF'IQ | F'IQ | *∆*(*argF-lacZ*)*U169 phoA glnV44 purB*(*E115K*) *ø80d* Δ(*lacZ*)*M15 gyrA96 recA1 endA1 relA1 thiE1 hsdR17* |  | Bethesda Research Labs |
| ER2884 |  |  | *rph-1* | pKD20 | ER1506 X pKD20 → ApR @ 30 ^o^C |
| ER2895 |  | - | *rph-1* Δ(*yjiT-mrr*)::FRT::*npt*(KnR) |  | ER2884 X PCR product pKD4 Δ(yjiT-mrr) → KnR |
| ER3155 |  | - | *fhuA2*::IS2 Δ(*lacZ*)*4826 glnX44 trpE31* Δ(*hisG*)*1 recD1014 rpsL104*(*StrR*) *xyl-7 mtlA2*(*Fs*) *metB1*(*FS*) Δ(*yjiT-mrr*)::FRT::*npt*(*KnR*) *serB28* |  | ER1636 X P1*vir*(ER2895) → KnR (Ser- EcoK-) |
| ER3164 |  | - | *fhuA2*::IS2 Δ(*lacZ*)*4826 glnX44 trpE31* Δ(*hisG*)*1 srl1300*::*Tn10* (*TetR*) *recD1014 rpsL104*(*StrR*) *xyl-7 mtlA2*(*Fs*) *metB1*(*FS*) Δ(*yjiT-mrr*)::FRT::*npt*(*KnR*) *serB28* |  | ER3155 X P1*vir*(ER2253) → TcR (Srl- RecA+) |
| ER3252 | SL13629 | - | *∆*(*araBAD*)*567 ∆lacZ4787*(::*rrnB3*) *∆recA*::FRT::*npt*(*KnR*) *rph-1 ∆*(*rhaBAD*)*568 hsdR514* |  | Susan Lovett [9] |
| ER3259 |  | - | *fhuA2*::IS2 Δ(*lacZ*)*4826 glnX44 trpE31* Δ(*hisG*)*1 gyrA96*(*NlR*) *srl1300*::*Tn10*(*TetR*) *recD1014 rpsL104*(*StrR*) *xyl-7 mtlA2*(*Fs*) *metB1*(*FS*) Δ(*yjiT-mrr*)::FRT::*npt*(*KnR*) *serB28* |  | ER3164 X P1*vir*(ER2204)→ NlR (Ser- Srl- SmR Hsd- McrBC- McrA+) |
| ER3263 |  | - | *fhuA2*::IS2 Δ(*lacZ*)*4826 glnX44 trpE31* Δ(*hisG*)*1 gyrA96*(*NlR*) *recA13 recD1014 rpsL104*(*StrR*) *xyl-7 mtlA2*(*Fs*) *metB1*(*FS*) Δ(*yjiT-mrr*)::FRT::*npt*(*KnR*) *serB28* |  | ER3259 X P1*vir*(ER1607) → Srl+ (TcS Ser- His- Trp- Met- NlR UVS KnR) |
| ER3265 |  | HfrPO1 (*∆(pifA-yddA*) | *supQ80 e14-* (*mcrA-*) *relA1 spoT1 thiE1* | pCD2 | ER1954 X pCD2 → CmR 30 ^o^C |
| ER3268 |  | HfrPO1 (*∆(pifA-yddA*) | *supQ80 e14-* (*mcrA-*) *relA1 spoT1 thiE1 mrr*::(*pCD2 cat rep-ts tetA tetR*) |  | ER3265 → TcR CmR 42 ^o^C x2 purifications |
| ER3270 |  | HfrPO1 (*∆(pifA-yddA*) | *supQ80 e14-* (*mcrA-*) *relA1 spoT1 thiE1 mrr*::*tetAR* |  | ER3268 → ApR with CmR arrest x2 purifications → screened for TcR (CmS) @ 30 ^o^C |
| ER3271 |  | HfrPO1 (*∆(pifA-yddA*) | *supQ80 e14-* (*mcrA-*) *∆recA*::FRT::*npt*(*KnR*) *relA1 spoT1 thiE1 mrr*::*tetAR* |  | ER3268 X P1*vir*(ER3252) → KnR |
| ER3274 |  | HfrPO1 (*∆(pifA-yddA*) | *supQ80 e14-* (*mcrA-*) *∆recA*::FRT::*npt*(KnR) *relA1 spoT1 thiE1 mrr*::*tetAR* | pCP20 | ER3271 X pCP20 → ApR CmR @ 30 ^o^C |
| ER3276 |  | HfrPO1 (*∆(pifA-yddA*) | *supQ80 e14-* (*McrA-*) *∆recA*::FRT *relA1 spoT1 thiE1 mrr*::*tetAR* |  | ER3274 → TcR 42 ^o^C x2 purifications (CmS ApS) |
| ER3278 |  | F' (*∆(pifA-yddA*)*mrr::tetAR* | *fhuA2*::IS2 Δ(*lacZ*)*4826 glnX44 trpE31* Δ(*hisG*)*1 gyrA96 recA13 recD1014 rpsL104*(*StrR*) *xyl-7 mtlA2*(*Fs*) *metB1*(*FS*) *mrr*::*tetAR serB28?* |  | ER3276 X ER3263 → NlR @ 37 ^o^C (Cross 4) |
| ER3282 |  | F' (*∆(pifA-yddA*) *mrr::tetAR* | *fhuA2*::IS2 Δ(*lacZ*)*4826 glnX44 trpE31* Δ(*hisG*)*1 gyrA96 recA13 recD1014 rpsL104*(*StrR*) *xyl-7 mtlA2*(*Fs*) *metB1*(*FS*) Δ(*yjiT-mrr*)::*npt*(KnR) *serB28* |  | ER3276 X ER3263 → NlR @ 37 ^o^C (Cross 4) |
| ER3290 |  | F' (*∆(pifA-yddA*) *mrr::tetAR* | *fhuA2*::IS2 Δ(*lacZ*)*4826 glnX44 trpE31* Δ(*hisG*)*1 gyrA96 recA13 recD1014 rpsL104*(*StrR*) *xyl-7 mtlA2*(*Fs*) *metB1*(*FS*) Δ(*yjiT-mrr*)::*npt*(KnR) *serB28* |  | ER3276 X ER3263 → NlR @ 37 ^o^C (Cross 4) |
| ER3291 |  | F' (*∆(pifA-yddA*) *mrr::tetAR* | *fhuA2*::IS2 Δ(*lacZ*)*4826 glnX44 trpE31* Δ(*hisG*)*1 gyrA96 recA13 recD1014 rpsL104*(*StrR*) *xyl-7 mtlA2*(*Fs*) *metB1*(*FS*) *mrr*::*tetAR serB28?* |  | ER3276 X ER3263 → NlR @ 37 ^o^C (Cross 4) |
| ER3333 |  | - | *fhuA2*::IS2 Δ(*lacZ*)*4826 glnX44 trpE31* Δ(*hisG*)*1 gyrA96 rpsL104*(*StrR*) *recA13 recD1014 xyl-7 mtlA2*(*Fs*) *mTn7*(Φ (*rhaBp-yjiA*) *metB1*(*FS*) Δ(*yjiT-mrr*)::FRT::*npt*(*KnR*) *serB28* |  | ER3263 X pER456 → ApR @ 42 ^o^C x2 purifications → screened for insertion at *attTn7* |
| ER3336 |  | - | *fhuA2*::IS2 Δ(*lacZ*)*4826 glnX44 trpE31* Δ(*hisG*)*1 gyrA96 recA13 recD1014 rpsL104*(*StrR*) *xyl-7 mtlA2*(*Fs*) *mTn7*(Φ (*rhaBp-yjiP*)) *metB1*(*FS*) Δ(*yjiT-mrr*)::FRT::*npt*(*KnR*) *serB28* |  | ER3263 X pER452 → ApR @ 42 ^o^C x2 purifications → screened for insertion at attTn7 |
| ER3340 |  | - | *fhuA2*::IS2 Δ(*lacZ*)*4826 glnX44 trpE31* Δ(*hisG*)*1 gyrA96 recA13 recD1014 rpsL104*(*StrR*) *xyl-7 mtlA2*(*Fs*) *mTn7*(Φ (*rhaBp-lacZ*)) *metB1*(*FS*) Δ(*yjiT-mrr*)::FRT::*npt*(*KnR*) *serB28* |  | ER3263 X pMS34 → ApR @ 42 ^o^C x2 purifications → screened for insertion at *attTn7* |
| ER3420 |  | HfrPO1 (*∆(pifA-yddA*) | *supQ80 thi-1 e14-* (*McrA-*) *∆recA*::FRT *relA1 spoT1 mrr*::*tetAR* | pKD46 | ER3276 X pKD46 → ApR TcR @ 30 ^o^C |
| ER3431 |  | HfrPO1 (*∆(pifA-yddA*) *∆*(*ybiB-yfgA)(::npt::*FRT) | *supQ80 thi-1 e14-* (*McrA-*) *∆recA*::FRT *relA1 spoT1 mrr*::(*tetA tetR*) |  | ER3420 X *ybiB*-P1-*npt*(FRT)-P2-oriT PCR product → KnR TcR @ 42 ^o^C |
| ER3433 |  | - | *fhuA2*::IS2 Δ(*lacZ*)*4826 glnX44 trpE31* Δ(*hisG*)*1 gyrA96 recA13 recD1014 rpsL104*(*StrR*) *xyl-7 mtlA2*(*Fs*) *metB1*(*FS*) Δ(*yjiT-mrr*)::FRT::*npt*(*KnR*) *serB28* | pKD46 | ER3263 X pKD46 → ApR NlR KnR SmR @ 30 ^o^C |
| ER3434 |  | HfrPO1 (*∆(pifA-yddA*)*∆(ybiB-yfgA)(::npt::FRT)* | *supQ80 thi-1 e14-* (*McrA-*) *∆recA*::FRT *relA1 spoT1 mrr*::(*tetA tetR*) | pCP20 | ER3431 X pCP20 → ApR CmR @ 30 ^o^C |
| ER3435 |  | HfrPO1 (*∆(pifA-yddA*) *∆*(*ybiB-yfgA*)(::FRT) | *supQ80 thiE1 e14-* (*McrA-*) *∆recA*::FRT *relA1 spoT1 mrr*::*tetAR* |  | ER3434 → TcR ApS CmS KnS @ 42 ^o^C x2 purifications |
| ER3436 |  | - | *fhuA2*::IS2 Δ(*lacZ*)*4826 glnX44 trpE31* Δ(*hisG*)*1 gyrA96 recA*::*cat*(FRT)(CmR) *recD1014 rpsL104*(*StrR*) *xyl-7 mtlA2*(*Fs*) *metB1*(*FS*) Δ(*yjiT-mrr*)::*npt*(KnR) *serB28* |  | ER3433 X *recA*-P1-cat(FRT)-P2-*recA* → CmR @ 37 ^o^C → CmR KnR NlR SmR @ 42 ^o^C |
| ER3439 |  | - | *fhuA2*::IS2 Δ(*lacZ*)*4826 glnX44 trpE31* Δ(*hisG*)*1 gyrA96 rpsL104*(*StrR*) *recA13 recD1014 xyl-7 mtlA2*(*Fs*) *mTn7*(Φ (*rhaBp-yjiP*) *metB1*(*FS*) Δ(*yjiT-mrr*)::*npt*(*KnR*) *serB28* | pKD46 | ER3336 X pKD46 → ApR @ 30 ^o^C |
| ER3440 |  | - | *fhuA2*::IS2 Δ(*lacZ*)*4826 glnX44 trpE31* Δ(*hisG*)*1 gyrA96 recA*::*cat*(FRT)(CmR) *recD1014 rpsL104*(*StrR*) *xyl-7 mtlA2*(*Fs*) *metB1*(*FS*) Δ(*yjiT-mrr*)::*npt*(KnR) *serB28* |  | ER3433 + *recA*-P1-*cat*(FRT)-P2-*recA* PCR product → CmR @ 37 ^o^C → CmR KnR NlR SmR @ 42 ^o^C |
| ER3445 |  | - | *supQ80?* Δ(*hisG*)*1 e14-* (*McrA-*) *gyrA96 recA*::*cat*(FRT)(CmR) *recD1014 rpsL104*(*StrR*) *xyl-7 mtlA2*(*Fs*) *metB1*(*FS*) *mrr*::*tetAR* |  | ER3440 X ER3435 → TcR NlR SmR CmR (Cross 6) |
| ER3446 |  | - | *supQ80?* Δ(*hisG*)*1 e14-* (*McrA-*) *gyrA96 recA*::*cat*(FRT)(CmR) *recD1014 rpsL104*(*StrR*) *xyl-7 mtlA2*(*Fs*) *metB1*(*FS*) *mrr*::*tetAR* |  | ER3440 X ER3435 → TcR NlR SmR CmR (Cross 6) |
| ER3454 |  | - | *fhuA2*::IS2 Δ(*lacZ*)*4826 glnX44 trpE31* Δ(*hisG*)*1 gyrA96 recA*::*cat*(FRT)(CmR) *recD1014 rpsL104*(*StrR*) *xyl-7 mtlA2*(*Fs*) *metB1*(*FS*) *mrr*::*tetAR serB28* |  | ER3440 X ER3435 → TcR NlR SmR CmR (Cross 6) |
| ER3455 |  | - | *fhuA2*::IS2 Δ(*lacZ*)*4826 glnX44 trpE31* Δ(*hisG*)*1 gyrA96 recA*::*cat*(FRT)(CmR) *recD1014 rpsL104*(*StrR*) *xyl-7 mtlA2*(*Fs*) *metB1*(*FS*) *[DUP*((*rrsB-rrlH*) (*yjiT-mrr*)::*npt*(*KnR*) *mrr*::*tetAR*)] *serB28* |  | ER3440 X ER3435 → TcR NlR SmR CmR (Cross 6) |
| ER3460 |  | - | *fhuA2*::IS2 Δ(*lacZ*)*4826 glnX44 trpE31* Δ(*hisG*)*1 gyrA96 recA*::*cat*(FRT)(CmR) *recD1014 rpsL104*(*StrR*) *xyl-7 mtlA2*(*Fs*) *mTn7*(Φ(*rhaBp-yjiP*)) *metB1*(*FS*) Δ(*yjiT-mrr*)::FRT::*npt*(*KnR*) *serB28* |  | ER3439 X recA-P1-*cat(FRT)*-P2-*recA* PCR product → CmR @ 37 ^o^C → CmR KnR NlR SmR @ 42 ^o^C |
| ER3461 |  | - | *fhuA2*::IS2 Δ(*lacZ*)*4826 glnX44 trpE31 hisG1 recA*::*kan*(FRT) (KnR) *recD1014 rpsL104*(*StrR*) *xyl-7 mtlA2*(*Fs*) *metB1*(*FS*) *serB28* |  | ER1636 X P1vir(ER3252) → KnR @ 37 ^o^C |
| ER3462 |  | - | *fhuA2*::IS2 Δ(*lacZ*)*4826 glnX44 trpE31* Δ(*hisG*)*1 recD1014 rpsL104*(*StrR*) *xyl-7 mtlA2*(*Fs*) *metB1*(*FS*) *serB28* | pKD46 | ER1636 X pKD46 → ApR 30 ^o^C (SmR) |
| ER3463 |  | - | *fhuA2*::IS2 Δ(*lacZ*)*4826 glnX44 trpE31* Δ(*hisG*)*1 recA*::*kan*(FRT) *recD1014 rpsL104*(*StrR*) *xyl-7 mtlA2*(*Fs*) *metB1*(*FS*) *serB28* | PCP20 | ER3461 X pCP20 → ApR @ 30 ^o^C |
| ER3464 |  | - | *fhuA2*::IS2 Δ(*lacZ*)*4826 glnX44 trpE31* Δ(*hisG*)*1 recD1014 rpsL104*(*StrR*) *xyl-7 mtlA2*(*Fs*) *metB1*(*FS*) *serB28 recA*::FRT |  | ER3463 → SmR @ 42 ^o^C x2 purifications |
| ER3466 |  | - | *supQ80* Δ(*hisG*)*1 gyrA96 e14-* (*McrA-*) *rpsL104*(*StrR*) *recA*::*cat*(FRT) *recD1014 xyl-7 mtlA2*(*Fs*) *thiE1 mrr*::*tetAR* |  | ER3460 X ER3435 → TcR NlR SmR CmR @ 37 ^o^C (cross 13) |
| ER3467 |  | - | *fhuA2*::IS2 Δ(*lacZ*)*4826 glnX44 trpE31* Δ(*hisG*)*1 recA*::FRT *recD1014 rpsL104*(*StrR*) *xyl-7 mtlA2*(*Fs*) *metB1*(*FS*) *serB28* | pKD46 | ER3464 X pKD46 → ApR @ 30 ^o^C |
| ER3468 |  | - | *fhuA2*::IS2 Δ(*lacZ*)*4826 glnX44 trpE31* Δ(*hisG*)*1 recD1014 rpsL104*(*StrR*) *xyl-7 mtlA2*(*Fs*) *metB1*(*FS*) *yjiT*::*npt*(FRT) *serB28* | pKD46 | ER3462 X *yjiT*-P1-*npt*(FRT)-P2-*yjiT* PCR product → KnR @ 30 ^o^C |
| ER3469 |  | - | *fhuA2*::IS2 Δ(*lacZ*)*4826 glnX44 trpE31* Δ(*hisG*)*1 recA*::FRT *recD1014 rpsL104*(*StrR*) *xyl-7 mtlA2*(*Fs*) *metB1*(*FS*) *yjiT*::*npt*(FRT) *serB28* | pKD46 | ER3467 X *yjiT*-P1-*npt*(FRT)-P2-*yjiT* PCR product → KnR @ 30 ^o^C |
| ER3472 |  | - | *fhuA2*::IS2 Δ(*lacZ*)*4826 glnX44 trpE31* Δ(*hisG*)*1 recD1014 rpsL104*(*StrR*) *xyl-7 mtlA2*(*Fs*) *metB1*(*FS*) *yjiP*::*cat*(FRT) *yjiT*::*npt*(FRT) *serB28* |  | ER3468 + *yjiT*-P1-*npt*(FRT)-P2-*yjiT* PCR product → CmR @ 37 ^o^C, purify x2 CmR KnR SmR @ 42 ^o^C |
| ER3473 |  | - | *fhuA2*::IS2 Δ(*lacZ*)*4826 glnX44 trpE31* Δ(*hisG*)*1 recA*::FRT *recD1014 rpsL104*(*StrR*) *xyl-7 mtlA2*(*Fs*) *metB1*(*FS*) *yjiP*::*cat*(FRT) *yjiT*::*npt*(FRT) *serB28* |  | ER3468 X *yjiP*-P1-*cat*(FRT)-P2-*yjiP* PCR product → CmR @ 37 ^o^C, purify x2 CmR KnR SmR @ 42 ^o^C |
| ER3475 |  | HfrPO1 (*Δ(pifA-yddA*) *∆*(*ybiB-yfgA*::FRT) | *supQ80* Δ(*hisG*)*1 e14-* (*McrA-*) *gyrA96 recA*::*cat*(FRT) *recD1014 rpsL104*(*StrR*) *xyl-7 mtlA2*(*Fs*) *metB1*(*FS*) *mrr*::*tetAR* |  | ER3460 X ER3435 → TcR NlR SmR CmR @ 37 ^o^C (cross 13) |
| ER3476 |  | - | *fhuA2*::IS2 Δ(*lacZ*)*4826 glnX44 trpE31* Δ(*hisG*)*1 gyrA96 recA*::*cat*(FRT) *recD1014 rpsL104*(*StrR*) *xyl-7 mtlA2*(*Fs*) *metB1*(*FS*) *mrr*::*tetAR* |  | ER3460 X ER3435 → TcR NlR SmR CmR @ 37 ^o^C (cross 13) |
| ER3480 |  | - | *fhuA2*::IS2 Δ(*lacZ*)*4826 glnX44 trpE31* Δ(*hisG*)*1 recD1014 rpsL104*(*StrR*) *xyl-7 mtlA2*(*Fs*) *mTn7*(Φ(*rhaBp-yjiP*) *metB1*(*FS*) *yjiP*::*cat*(FRT) *yjiT*::*npt*(FRT) *serB28* |  | ER3472 X pER452 → ApR @ 37 ^o^C → SmR @ 42 ^o^C x2 purifications → screened for insertion at attTn7 |
| ER3481 |  | - | *fhuA2*::IS2 Δ(*lacZ*)*4826 glnX44 trpE31* Δ(*hisG*)*1 recD1014 rpsL104*(*StrR*) *xyl-7 mtlA2*(*Fs*) *mTn7*(Φ(*rhaBp-yjiP*) *metB1*(*FS*) *serB28 recA*::FRT *yjiT*::*npt*(FRT) *yjiP*::*cat*(FRT) |  | ER3473 X pER452 → ApR @ 37 ^o^C → SmR @ 42 ^o^C x2 purifications → screened for insertion at *attTn7* |
| ER3482 |  | - | *fhuA2*::IS2 Δ(*lacZ*)*4826 glnX44 trpE31* Δ(*hisG*)*1 recA*::FRT *recD1014 rpsL104*(*StrR*) *xyl-7 mtlA2*(*Fs*) *metB1*(*FS*) *yjiP*::*cat*(FRT) *mrr*::*tetAR serB28* |  | ER3435 X ER3473 →TcR SmR (Cross 8) |
| ER3483 |  | - | *fhuA2*::IS2 Δ(*lacZ*)*4826 glnX44 trpE31* Δ(*hisG*)*1 recA*::FRT *recD1014 rpsL104*(*StrR*) *xyl-7 mtlA2*(*Fs*) *metB1*(*FS*) *yjiP*::*cat*(FRT) *mrr*::*tetAR serB28* |  | ER3435 X ER3473 →TcR SmR (Cross 8) |
| ER3484 |  | - | *fhuA2*::IS2? Δ(*lacZ*)*4826? glnX44? trpE31?* Δ(*hisG*)*1? recA*::FRT *recD1014 rpsL104*(*StrR*) *xyl-7 mtlA2*(*Fs*) *metB1*(*FS*) *yjiP*::*cat*(FRT) *mrr*::*tetAR serB28?* |  | ER3435 X ER3473 →TcR SmR (Cross 8) |
| ER3510 |  | - | *fhuA2*::IS2 Δ(*lacZ*)*4826 glnX44 trpE31* Δ(*hisG*)*1 recA*::FRT *recD1014 rpsL104*(*StrR*) *xyl-7 mtlA2*(*Fs*) *metB1*(*FS*) *yjiP*::*cat*(FRT) *mrr*::*tetAR serB28* |  | ER3435 X ER3473 →TcR SmR (Cross 8) |
| ER3544 |  | - | *∆(argF-lac)U169 glnX44 mcr-67 rfbD1? relA1? endA1 spoT1? dinD2::MudI1734 (KanR, lacZ(ts)) thi-1 ∆([fimB or yjiT]-opgB)114::IS10* | pER452 | ER2170 X pER452 🡪 ApR @ 30 ^o^C |

^a^All strains in this list are derived from *E. coli* K12

| **Vector** | **Genotype** | **Source** |
| --- | --- | --- |
| pMS34 | *oripSC101ts ApR araBp*::*TnsABCD araCp-araC mTn7*(Φ(*rhaBp-lacZ*) | [10] |
| pMAK705 | *oripSC101ts bla* (ApR) *cat* (CamR) *lacZ* | [11] |
| pCP20 | *ts-rep* [cI857](λ)(ts) *bla*(ApR) *cat* FLP([]) | [12] |
| pKD20 | *repA101*(*ts*) *araBp-gam-bet-exo oriR101 bla*(ApR) *araC bla* [tL3] | [12] |
| pKD46 | *repA101*(*ts*) *araBp-gam-bet-exo oriR101 bla*(ApR) *araC bla* [tL3] | [12] |
| pKD4 | *oriR*(R6Kγ) *bla*(AmpR) *rgnB*(Ter) *npt*(KnR) | [12] |
| pKD32 | *oriR*(R6Kγ) *tL3*λ(*ter*) *bla*(AmpR) *rgnB*(Ter) *cat*(CamR) | [12] |
| pTK013 | pMAK705 pIS2-*npt*-*oriT* | this work |
| pER452 | PMS34 *mTn7*(Φ(*rhaBp-yjiP*) | this work |
| pER456 | PMS34 *mTn7*(Φ(*rhaBp-yjiA*) | this work |
| pCD2 | *lac*(α)::*mrr*::(*tetAtetR*) *cat* (CmR) *rep*(*pSC101ts*) | this work |

| **Oligo #** | **Name** | **Sequence** |
| --- | --- | --- |
| oTK021 | pIS2(comp)_fwd | AGTGAATTCGAGCTCGGTACTAATGTGGAGCTGGAAAAATG |
| oTK022 | pIS2(comp)_rev | TCGAAGCAGCAGAAATTTCACATGACTGC |
| oTK023 | kan(FLP)_fwd | TGAAATTTCTGCTGCTTCGAAGTTCCTATAC |
| oTK024 | kan(FLP)_rev (gib) | GAACCCGCTGATGGGAATTAGCCATGGTC |
| oTK025 | poriT(comp)_fwd | TAATTCCCATCAGCGGGTTCAGGAGAATATG |
| oTK026 | poriT(comp)_rev | GCCAAGCTTGCATGCCTGCATGCCACTGATTGCTTTGATC |
| oTK033 | recA-up-P1 | CAGAACATATTGACTATCCGGTATTACCCGGCATGACAGGAGTAAAAATGTGTAGGCTGGAGCTGCTT |
| oTK034 | recA-do-P2 | ATGCGACCCTTGTGTATCAAACAAGACGATTAAAAATCTTCGTTAGTTTCCTGACATGGGAATTAGCCAT |
| oTK037 | yjiP_up_fwd | TCAAAAGAGCGCGGTGGA |
| oTK038 | yjiP_up_rev (cas) | GCCTACACAAGGTTTTAAATAATGCATCATGCG |
| oTK039 | cassette_fwd (yjiP) | TTAAAACCTTGTGTAGGCTGGAGCTGCT |
| oTK040 | cassette_rev (yjiP) | CCAGTAATAGACATGGGAATTAGCCATGG |
| oTK041 | yjiP_do_fwd (cas) | CCCATGTCTATTACTGGTTACAGAGTGTG |
| oTK042 | yjiP_do_rev | GTAATCTTTTGTATCCATTCAGG |
| oTK043 | yjiT_up_fwd | TGAATATACCCAATTAACGCAG |
| oTK044 | yjiT_up_rev (cas) | GCCTACACAAACCACCTTCACTGAATAATG |
| oTK045 | cassette_fwd (yjiT) | GGTGGTTTGTGTAGGCTGGAGCTGCT |
| oTK046 | cassette_rev (yjiT) | GTACTCTTATGACATGGGAATTAGCCATGG |
| oTK047 | yjiT_do_fwd (cas) | CCCATGTCATAAGAGTACGCGTTGCC |
| oTK048 | yjiT_do_rev | TGCTCCCAACAATCATCC |
| pER73 | UPyjiS | GAACTGCCAGAAGGTGGATG |
| pER74 | DNyjiA | GAGTCGGCGGATAAACTGCTA |
| pER89 | mrrrightTetR | ACGGCATCCGGCCGTGACACGTTGGAGCCGCATTATTTTCGC |
| pER90 | mrrleftTetA | GAGGCTGCCGCGGATGCAACCGCGGCTTTTTATTGAGCTTGA |
| pER91 | pMAKmrrright | GCTCGGTACCCGGGGATCCAGGCCGTGGGGCGATGAAA |
| pER92 | pMAKmrrleft | TACGCCAAGCTTGCATGCCATTGCTGTGCGGGCCTGTCC |
| pER93 | tetRmrrright (anti 89) | GCGAAAATAATGCGGCTCCAACGTGTCACGGCCGGATGCCGT |
| pER94 | tetAmrrleft (anti 90) | TCAAGCTCAATAAAAAGCCGCGGTTGCATCCGCGGCAGCCTC |
| prER126 | yjiP5'_fwd | ACACAGGAGGGACGTCATGACAAACTTCACGACCAG |
| prER127 | yjiP5'_rev | GCTCTATATGGCGCTGCATC |
| prER128 | yjiP3'_fwd | TCCATGGCGGTGATGCAG |
| prER129 | yjiP3'_rev | CCAGCGGCCGCGTTTAAACTAGCTCTTGAGCCATGAATAGC |
| prER130 | Tn7L flanking primer | CATGGCAATTCTGGAAGAAATAG |
| prER131 | Tn7R flanking primer | GATCTAAACTATGACAATAAAG |
| prER132 | attTn7down | GATGACGGTTTGTCACATGGA |
| prER133 | attTn7up | GATGCTGGTGGCGAAGCTGT |
| prER136 | yjiA_fwd | ACACAGGAGGGACGTCATGAACCCGATTGCAGTTAC |
| prER137 | yjiA_rev | CCAGCGGCCGCGTTTAAACTTACTTCCTCAACCCCGC |

1. Raleigh EA, Wilson G (1986) Escherichia coli K-12 restricts DNA containing 5-methylcytosine. Proc Natl Acad Sci U S A 83: 9070-9074.

2. Bachmann BJ (1987) Derivations and genotypes of some mutant derivatives of *Escherichia coli* K-12. In: Neidhardt FC, Ingraham JL, Low KB, Magasanik B, Schaechter M et al., editors. Escherichia coli and Salmonella typhimurium: Cellular and molecular biology. Washington, D.C.: American Society for Microbiology. pp. 1190-1219.

3. Hill CW, Harnish BW (1981) Inversions between ribosomal RNA genes of Escherichia coli. Proc Natl Acad Sci U S A 78: 7069-7072.

4. Jensen KF (1993) The Escherichia coli K-12 "wild types" W3110 and MG1655 have an rph frameshift mutation that leads to pyrimidine starvation due to low pyrE expression levels. J Bacteriol 175: 3401-3407.

5. Guyer MS, Reed RR, Steitz JA, Low KB (1981) Identification of a sex-factor-affinity site in E. coli as gamma delta. Cold Spring Harb Symp Quant Biol 45 Pt 1: 135-140.

6. Amundsen SK, Taylor AF, Chaudhury AM, Smith GR (1986) recD: the gene for an essential third subunit of exonuclease V. Proc Natl Acad Sci U S A 83: 5558-5562.

7. Raleigh EA, Murray NE, Revel H, Blumenthal RM, Westaway D, et al. (1988) McrA and McrB restriction phenotypes of some E. coli strains and implications for gene cloning. Nucleic Acids Res 16: 1563-1575.

8. Piekarowicz A, Yuan R, Stein DC (1991) A new method for the rapid identification of genes encoding restriction and modification enzymes. Nucleic Acids Res 19: 1831-1835.

9. Baba T, Ara T, Hasegawa M, Takai Y, Okumura Y, et al. (2006) Construction of Escherichia coli K-12 in-frame, single-gene knockout mutants: the Keio collection. Mol Syst Biol 2: 2006 0008.

10. Sibley MH, Raleigh EA (2012) A versatile element for gene addition in bacterial chromosomes. Nucleic Acids Res 40: e19.

11. Hamilton CM, Aldea M, Washburn BK, Babitzke P, Kushner SR (1989) New method for generating deletions and gene replacements in Escherichia coli. J Bacteriol 171: 4617-4622.

12. Datsenko KA, Wanner BL (2000) One-step inactivation of chromosomal genes in Escherichia coli K-12 using PCR products. Proc Natl Acad Sci U S A 97: 6640-6645.
