# Supplementary material for: Novel recA-Independent Horizontal Gene Transfer in Escherichia coli K-12
Source: PLoS One. 2015 Jul 10;10(7):e0130813. doi: 10.1371/journal.pone.0130813 (PMC4498929; doi:10.1371/journal.pone.0130813)
Supplement: S2 Table — (DOCX) [file pone.0130813.s009.docx]

**S2 Table. Mating partners and conditions used in this study.^a^**

| **Cross ref #** | **Donor Strain** | **Recipient Strain** | **Mating Duration (h)** | **Recombinant selection (μg/mL)^b^** | | | | **Figure(s)** |
| --- | --- | --- | --- | --- | --- | --- | --- | --- |
|  |  |  |  | **Sm** | **Tc** | **Nl** | **Cm** |  |
| 1 | ER3270 | ER1636 | 0.25 | 100 | 20 |  |  | 3 |
| 2 | ER3276 | ER1636 | 0.25 | 100 | 20 |  |  | 3 |
| 3 | ER3435 | ER1636 | 0.25 | 100 | 20 |  |  | 3 |
| 4 | ER3276 | ER3263 | 0.25 | 50 | 10 | 5 |  | 3, S1, S6 |
| 5 | ER3435 | ER3436 | 18 | 50 | 10 | 5 | 15 | 3, 4 |
| 6 | ER3435 | ER3440 | 18 | 50 | 10 | 5 | 15 | 5, S4-6 |
| 7 | ER3435 | ER3472 | 0.25 | 100 | 20 |  |  | 6 |
| 8 | ER3435 | ER3473 | 18 | 100 | 20 |  |  | 6, |
| 9 | ER3435 | ER3480 | 0.25 | 100 | 20 |  |  | S3 |
| 10 | ER3435 | ER3481 | 18 | 100 | 20 |  |  | 6 |
| 11 | ER3276 | ER3333 | 0.25 | 50 | 10 | 5 |  | S3 |
| 12 | ER3276 | ER3336 | 0.25 | 50 | 10 | 5 |  | S3 |
| 13 | ER3435 | ER3460 | 18 | 50 | 10 | 5 | 15 | 4, S3 |

^a^Each cross is listed, with strain names, duration, drug selections and where the results are described.

^b^Antibiotic abbreviations: Sm (streptomycin), Tc (tetracycline), Nl (nalidixic acid), and Cm (Chloramphenicol).
