# Supplementary material for: Novel recA-Independent Horizontal Gene Transfer in Escherichia coli K-12
Source: PLoS One. 2015 Jul 10;10(7):e0130813. doi: 10.1371/journal.pone.0130813 (PMC4498929; doi:10.1371/journal.pone.0130813)
Supplement: S3 Table — (DOCX) [file pone.0130813.s010.docx]

**S3 Table Genes and relevant features within crossover intervals of the large patch RecA-independent genomic replacement recombinants.^a^**

| *Proximal Crossover Interval* |  | ER3445 (605 bps) | |  | ER3446, ER3476 (9639 bps) | |  | ER3454 (3686 bps) | |  | ER3466 (3515bps) | |  | ER3475 (9530 bps) | |  |
| --- | --- | --- | --- | --- | --- | --- | --- | --- | --- | --- | --- | --- | --- | --- | --- | --- |
|  |  | Genes | Features |  | Genes | Features |  | Genes | Features |  | Genes | Features |  | Genes | Features |  |
|  |  | **IS1***/fimE*^b, c^ | mobile element,  recipient variation |  | distal *yjhP* | recipient SNP |  | **IS1***/fimE*^b^ | mobile element,  recipient variation |  | *bglB* | recipient SNP |  | *fimD* | recipient SNP |  |
|  |  |  |  |  | *yjhPQXZ* |  |  |  |  |  | *bglF* | Chi site |  | *fimFGH* | Chi site (fimH) |  |
|  |  | *fimS ^d^* | invertible,  donor variation |  | *yjhR* | Chi site |  | *fimS*^d^* | invertible,  unique variation |  | *bglG* | donor SNP |  | *gntP* |  |  |
|  |  |  |  |  | *nanSMC* |  |  |  |  |  |  |  |  | *uxuAB* |  |  |
|  |  |  |  |  | *fimB* | *fimS* invertase |  | *fimAIC* |  |  |  |  |  | *uxuR* | 2 Chi sites |  |
|  |  |  |  |  | IS1*/****fimE***^b^ | *fimS* invertase,  donor variation |  | *fimD* | donor SNP |  |  |  |  | ***yjiC****/*IS5^b^ | donor variation |  |
|  |  |  |  |  |  |  |  |  |  |  |  |  |  |  |  |  |

| Distal crossover interval |  | ER3445 (19570 bps) | |  | ER3446, ER3466, ER3475 (44501 bps) | |  | ER3454 (6454 bps) | |  | ER3476 (38150 bps) | |
| --- | --- | --- | --- | --- | --- | --- | --- | --- | --- | --- | --- | --- |
|  |  | Genes | features |  | Genes | Features |  | Genes | Features |  | Genes | Features |
|  |  | *isrA* | donor SNP |  | proximal *sra* | donor SNP |  | *fhuF* | donor SNP |  | *carB* | donor SNP, Chi site |
|  |  | *smrA* |  |  | *sra* |  |  | *yjjZ* |  |  | *caiFEDCBAT* | Chi site (*caiD*) |
|  |  | *ydaM* |  |  | *bdm* |  |  | *leuQP* |  |  | *fixABCX* | Chi sites (*fixA, C*) |
|  |  | *zntB* | Chi site |  | *osmC* |  |  | *rsmC* |  |  | *yaaU* |  |
|  |  | *fnrS* |  |  | *ddpFDCBAX* | Chi site (*ddpA*) |  | *holD* |  |  | *kefF* |  |
|  |  | *dbpA* |  |  | *dosPC* | Chi site (*dosP*) |  | *rimI* |  |  | *kefC* | 2 Chi sites |
|  |  | *ttcA* | Chi site & Rac^e^ |  | *yddW* | 3 Chi sites |  | *yjjG* |  |  | *folA* |  |
|  |  | *intR* | Rac^e^ |  | *gadCB* |  |  | *prfC* |  |  | *apaHG* |  |
|  |  | *ydaQC* | Rac^e^ |  | *pqqL* |  |  | *osmY* |  |  | *rsmA* |  |
|  |  | *lar* | Rac^e^ |  | *yddBA* |  |  | distal *osmY* | recipient SNP |  | *pdxA* |  |
|  |  | *recET* | Rac recombinase^e^ |  | *ydeMNO* |  |  |  |  |  | *surA* |  |
|  |  | *racC* | Rac^e^ |  | *safA* |  |  |  |  |  | *lptD* |  |
|  |  | *ydaF* | Rac^e^ |  | *ydePQRST* |  |  |  |  |  | *djlA* | Chi site |
|  |  | *kilR* | Rac^e^ |  | *yneL* |  |  |  |  |  | *yabP* |  |
|  |  | *sieB* | Rac^e^ |  | *dif* | XerCD recombinase  binding site |  |  |  |  | *rulA* |  |
|  |  | *ydaFG* | Rac^e^ |  |  |  |  |  |  |  | *rapA* | Chi site |
|  |  | *racR* | Rac^e^ |  | *hipAB* |  |  |  |  |  | *polB* | Chi site & DNA pol |
|  |  | *ydeSTUVW* | Rac^e^ |  | *yneO* |  |  |  |  |  | *araDABC* | Chi sites (*araA, B*) |
|  |  | *rzpR/rzoR* | Rac^e^ |  | *lsrK* |  |  |  |  |  | *yabI* | Chi site |
|  |  | *trkG* | Chi site & Rac^e^ |  | distal *lsrK* | recipient SNP |  |  |  |  | *thiQ* |  |
|  |  | distal *trkG* | recipient SNP |  |  |  |  |  |  |  | *thiP* | recipient SNP |

^a^ Analysis performed with the Geneious R7 visualization of Mauve alignment software.

^b^ For donor/recipient variations distinguished by different genes or mobile elements, the bolded feature indicates which was present in the recombinant.

^c^ See [1] for information on the IS1 mobile element.

*^d^ fimS* is an invertible segment present in different orientations in the donor and recipient strains [2]. In recombinant ER3454, *fimS* is a unique sequence.

^e^ Rac refers to a defective prophage integrated in the E. coli genome [3]. It contains the recET recombinase [4].

^f^ Xer-dif recombination usually facilitates the segregation of chromosome dimers during cell division [5]

1. Darmon E, Leach DR (2014) Bacterial genome instability. Microbiol Mol Biol Rev 78: 1-39.

2. McClain MS, Blomfield IC, Eisenstein BI (1991) Roles of fimB and fimE in site-specific DNA inversion associated with phase variation of type 1 fimbriae in Escherichia coli. J Bacteriol 173: 5308-5314.

3. Kolodner R, Hall SD, Luisi-DeLuca C (1994) Homologous pairing proteins encoded by the Escherichia coli recE and recT genes. Mol Microbiol 11: 23-30.

4. Muyrers JP, Zhang Y, Buchholz F, Stewart AF (2000) RecE/RecT and Redalpha/Redbeta initiate double-stranded break repair by specifically interacting with their respective partners. Genes Dev 14: 1971-1982.

5. Grainge I, Lesterlin C, Sherratt DJ (2011) Activation of XerCD-dif recombination by the FtsK DNA translocase. Nucleic Acids Res 39: 5140-5148.
