# Supplementary material for: Novel recA-Independent Horizontal Gene Transfer in Escherichia coli K-12
Source: PLoS One. 2015 Jul 10;10(7):e0130813. doi: 10.1371/journal.pone.0130813 (PMC4498929; doi:10.1371/journal.pone.0130813)
Supplement: S1 File — (DOCX) [file pone.0130813.s011.docx]

**Supplemental Methods**

**β-galactosidase assays**

Cultures of ER3340 containing the *rhaBp*-*lacZ* construct were grown to an OD_600_ 0.15 in RB. 0.2% rhamnose was added as appropriate, and growth continued at 37 ^o^C with continuous shaking. Samples were taken at rhamnose addition and then every 45 minutes for 6 hours. β-galactosidase assays were performed on the samples as described by Miller [1] except that cells were lysed with a lysozyme-nuclease-detergent solution for 10 minutes at 37 ^o^C instead of chloroform.

To test the effect of yjiP overexpression on the SOS response using the *dinB* reporter created by Piekarowicz [2], strain ER3544 (*rec*^+^ *rhaBp-yjiPc dinDp-lacZ*)*.* Was streaked on RB plates supplemented with 40 μg/μL X-gal (5-bromo-4-chloro-3-indolyl-β-D-galactopyranoside) and with or without 0.2% rhamnose. Plates were incubated at 30 ^o^C for 2 days and the color of the colonies were assessed visually.

**Illumina Sequencing**

Illumina MiSeq Nextera reads from Qiagen preps of donor and four recombinants were aligned with the donor reference sequence "ER3275_6 in silico assembly"*. Four recombinant genomes from the ER3276 x ER3263 matings were aligned individually with this reference using the Galaxy implementation of MIRA v3.4.0 [3] The resulting ACE files were imported into Tablet [4]. Screenshots of the visualizations were inverted and labeled. A) The entire genome alignment; B) the alignment for the region surrounding the transferred region in the recombinants. This is essentially a histogram of reads aligned with the reference. The even coverage over the majority of the genome (A) represents 1X coverage. Reads are absent for the F segment of the donor that is transferred last (no coverage in the recombinants, 1X coverage in the donor). The F-specific leading segment, from oriT to the position of IS2K (at which F integrated to form HfrH) exhibits ~2X coverage, consistent with expectation for an F-derived extrachromosomal plasmid [5,6]; except that even in the donor no reads align to the *oriS1* (*Δ(pifA-yddA*)), the second vegetative origin region of the original F plasmid. Apparently this origin was deleted in the establishment of HfrH. The region distal to the inserted F material is at higher coverage, consistent with the sum of 1X chromosomal material and 2X plasmid-borne copies; the ICR itself is similar to the F-specific segment, consistent with the absence of this segment from the recipient chromosome.

*The donor reference used was assembled in silico using MG1655 (NC_000913.2) and the F plasmid (AP001918) Genbank sequences as follows.

- - An *in silico* representation was created using Geneious (trial period; Biomatters, Ltd) and Lasergene SeqBuilder (DNAStar).
    - HfrH—F factor integration was imitated *in silico* using
  - The known sequence of the near-relative MG1655 (NC_000913.2)
  - The known sequence of the F factor AP001918 99159 bp
  - The known approximate position and orientation of the F-factor based on genetic mapping data using it [7]
  - The literature report concluding that Hfr formation is frequently due to homologous recombination between IS elements on the F and in the chromosome. [8]
  - A suitably-located IS2 element (*insC11insD11,* representing IS2K; EcoGene) is present in the MG1655 chromosome near the known approximate position of this integration event, and an IS2 element (represented by *ybiAybhB*) present on the F sequence.
- *tetRtetA* genes disrupting the *mrr* gene
  - created using SeqBuilder, an extracted segment of MG1655 as above and the sequence of Tn10 *tetRA* [9]
  - The in vivo verification of the *in silico* design of this disruption had been verified by PCR and Sanger sequencing of selected PCR products.
- loss of cryptic prophage e14
  - This was deleted from the sequence using published mechanistic and sequence data from the Campbell lab, which had studied the nature of excision events [10]
- Substitution of RecA CDS with an FRT sequence,
  - based on information in [11]

Numerous SNPs and IS variants were found when the descendant ER3435 was sequenced using Pacific Bioscience SMRT sequencing.

**Variant visualization using Geneious**

Pacific Biosciences RS_HGAP_assembly.3 de novo contigs were downloaded as fasta files, extracted contigs were imported into Geneious 7.1.7 (Biomatters, LTD), and named by ER strain number and contig (ERxx_0, ERxx_1 etc). These were annotated (Annotate and Predict menu) using an annotation database (folder) within the Geneious environment containing Genbank files of MG1655 (NC_00913.2), Plasmid F (AP001918), Tn10 Tet (J01830), Tn903 (V00621) and Tn9 (U63147) and with the PlasMapper database provided in the Geneious package. 100% nucleotide identity was required for annotation transfer, except that *tetA* has 3 changes to the canonical sequences (agrees with conflicts noted in the Genbank file). The primary contig in each case carried a terminal duplication due to assembly as a linear. This contig was trimmed to eliminate the duplication. Other contigs obtained usually were short, and contained one of the invertible segments of the genome in the other orientation than that of the main contig. These were easily identified by use of the annotations, and were not further analyzed. In some cases an extraneous plasmid replicon used as a sequencing control was erroneously included in the dataset, and was ignored.

The point of trimming was identified by extraction of the first and last segment for a contig that was at least twice as long as the longest Pacific Biosciences read. These terminal segments were aligned with each other using Mauve genome alignment within the Geneious environment, and the point of duplication identified. Half of the duplication was trimmed from one end, half from the other end of the original contig. This coordinate is most easily obtained from the Geneious Alignment view rather than the Mauve view. However, the Mauve view is useful if the terminal duplication includes one of the (at least) three invertible segments associated with cryptic prophages in K12. The HGAP assembler may put the reads with one orientation at one end and the reads with the other orientation at the other end instead of in a separate contig. In this case may show two LCBs, one inverted relative to the other.

The trimmed assembly was circularized by defining it as a circle (Sequence menu) and renumbered to obtain a common view. For most purposes, the origin was placed first upstream of *thrA* to agree with NC_00913.2 as follows: In sequence view, go to *thrA;* if *thrA* is on the bottom strand, choose "reverse complement" from the Sequence menu; save; obtain numerical position of *thrA* gene start=thrA#1*;* subtract 336 from thrA#1 =numerical_origin_to_be; Go To Base (Edit menu), enter numerical_origin_to_be (this puts the cursor on the base that will become the new origin); choose "Change Residue Numbering" (Sequence Menu), see that the number is what you want, check OK. For analysis of recombination patches, the numerical origin of all analyzed sequences was set to the first nucleotide of the DnaA coding sequence. In this way, patches that crossed the *thrA* numerical origin could be displayed as continuous patches on a linear map.

To identify and visualize variant positions, pairwise alignments of the two parents with each other and with each recombinant were made using Mauve (Align/assemble menus, "align whole genomes"). In alignment window, one parent was made the reference (highlight the sequence and choose "make reference"; save). If this isn't done, the bases of large indels will not be combined but will be listed separately. Variants were identified with "find SNPS/variations" in the Annotate and predict menu, with "merge adjacent variations" option. The variations can be exported as an Excel accessible file (.csv) or a screen visualization can be exported like the one in Fig. S5.

**References**

1. Miller JH (1972) Experiments in molecular genetics. Cold Spring Harbor, NY: Cold Spring Harbor Laboratory Press.

2. Piekarowicz A, Yuan R, Stein DC (1991) A new method for the rapid identification of genes encoding restriction and modification enzymes. Nucleic Acids Res 19: 1831-1835.

3. Chevreux B, Pfisterer T, Drescher B, Driesel AJ, Muller WE, et al. (2004) Using the miraEST assembler for reliable and automated mRNA transcript assembly and SNP detection in sequenced ESTs. Genome Res 14: 1147-1159.

4. Milne I, Bayer M, Cardle L, Shaw P, Stephen G, et al. (2010) Tablet--next generation sequence assembly visualization. Bioinformatics 26: 401-402.

5. Arutyunov D, Frost LS (2013) F conjugation: back to the beginning. Plasmid 70: 18-32.

6. Shizuya H, Birren B, Kim UJ, Mancino V, Slepak T, et al. (1992) Cloning and stable maintenance of 300-kilobase-pair fragments of human DNA in Escherichia coli using an F-factor-based vector. Proc Natl Acad Sci U S A 89: 8794-8797.

7. Wood TH (1968) Effects of temperature, agitation, and donor strain on chromosome transfer in Escherichia coli K-12. Journal of Bacteriology 96: 2077-2084.

8. Umeda M, Ohtsubo E (1989) Mapping of insertion elements IS1, IS2 and IS3 on the Escherichia coli K-12 chromosome. Role of the insertion elements in formation of Hfrs and F&apos; factors and in rearrangement of bacterial chromosomes. Journal of Molecular Biology 208: 601-614.

9. Bertrand KP, Postle K, Wray LV, Reznikoff WS (1983) Overlapping divergent promoters control expression of Tn10 tetracycline resistance. 149-156 p.

10. Wang H, Yang CH, Lee G, Chang F, Wilson H, et al. (1997) Integration specificities of two lambdoid phages (21 and e14) that insert at the same attB site. Journal of Bacteriology 179: 5705-5711.

11. Baba T, Ara T, Hasegawa M, Takai Y, Okumura Y, et al. (2006) Construction of Escherichia coli K-12 in-frame, single-gene knockout mutants: the Keio collection. Molecular Systems Biology 2: 2006.0008.
