# Supplementary material for: Novel recA-Independent Horizontal Gene Transfer in Escherichia coli K-12
Source: PLoS One. 2015 Jul 10;10(7):e0130813. doi: 10.1371/journal.pone.0130813 (PMC4498929; doi:10.1371/journal.pone.0130813)
Supplement: S2 File — (DOCX) [file pone.0130813.s012.docx]

**Validation and modification of the conjugal system**

Here we provide a full description of the mating configurations we tested to validate the conjugal system. We also detail and the conclusions derived from these experiments and explain how our findings led to further modifications of the system so we could focus on specific recombination events.

**Recombination proficient strains**

Our system can by validated by comparison of our homologous recombination efficiency to those reported in similar matings described in the literature. When our conjugal system was configured for homologous recombination (Cross 1 in Fig. 3A, *rec*^+^ donor and recipient), about 20% of the recipients acquired tetracycline resistance from the donor per hour of mating (recombination frequency 0.208 ± 0.032 recombinants/recipient per hour). This efficiency is comparable to the reported mating efficiencies in similar studies which usually range from 10% [1] to 30% [2,3] after 1-2 hours of mating.

An unexpected finding from our analysis of homologous recombination in this system was that the frequency of RecA-mediated exchange dropped four-fold when the *donor* was RecA-defective (to 0.053 ± 0.006 CFU/Rec^+^ recipient per hour; compare Crosses 1 and 2 Fig. 3A). Since RecA is not required for conjugal transfer and the recipient still has a functional *recA* gene for integrating foreign DNA, it is not immediately clear why deleting *recA* from the donor should affect recombination efficiency. Deleting *recA* modifies the physiology of the cell in many ways and any stage of conjugation, from pilus formation to DNA transfer, could be indirectly inhibited by these changes

**Episome recovery via microhomology-mediated circularization**

RecA-independent recombination from crossing a Δ*recA* donor with a *recA13* recipient (Cross 4, Fig. 3A) yielded recombinants at a frequency of (3.5 ± 1.1) X 10^-6^ CFU/recipient per hour. This ~10000-fold decrease in efficiency compared to the Rec^+^ of Cross 2 is similar to that observed by Low [4], who used *rec^+^* donors. However, phenotypic screens showed that over 80% of the recombinants were still KnR (Cross 4, Fig. 3B) suggesting that the donor’s tetracycline resistance cassette was often stably incorporated without replacing the corresponding locus on the recipient genome.

To determine the location of the stable copy of *tetAR*, we used Illumina sequencing to analyze the genomes of two KnR and two KnS recombinants (S2 Fig.). Short reads were aligned with an *in silico* model of the recipient and donor genome sequences. In each case, extra copies of donor chromosomal sequence were found; a continuous donor-homologous segment exhibited 2-4 fold-increased coverage compared to the rest of the sequence. This segment begins near *oriT* and ends at a distinct position beyond the ICR for each recombinant. *De novo* read assembly in each case yielded a contig in which the proximal donor end (*oriT* or a site 279 bp away in *ygfA*) is joined to a distal chromosomal site with 8 or 10 bases of identity to the proximal site. This suggests a circular donor segment in the recombinant, and a microhomology-mediated joining mechanism [5,6]. Collectively, these features are all consistent with independent F-prime (F’)-plasmid formation.

F’-plasmid formation in recombinants from the Rec-deficient mating is an unsurprising result, but the variety of chromosomal sites employed is unexpected. Low showed that DNA transferred by an Hfr strain can become a novel plasmid containing the F factor joined to chromosomal DNA in the recipient [7]. F'-plasmids carrying proximal markers were also found [8]; but in that case, preferred chromosomal sites related to *oriT* were employed for circularization. In the four recombinants we sequenced, each joining event occurred at a distinct locus on the chromosome that had not been reported previously

The ~17% of KnS recombinants from this cross were further analyzed for a *recA*-independent exchange of the recipient’s ICR. Phenotypic screens revealed that about half of the KnS recombinants were UV resistant (Fig. 3B). We infer that reversion of the *recA13* allele provides a small Rec^+^ recipient subpopulation, so that efficient homologous recombination of *mrr*::*tetAR* adds to the apparent frequency of *recA*-independent recombination in this system. We therefore completely deleted *recA* from the recipient for Cross 5, discussed above. No UV^R^ recombinants were obtained with a Δ*recA* recipient (Fig. 3B). The rest of the KnS recombinants from Cross 4 exhibited a UV^S^ phenotype, but were unstable to storage. We surmise that these result from formation of unstable F’-plasmids followed by additional RecA-independent recombination events that eliminated KnR. Segregational instability of the F'-plasmids would lead to toxicity due to loss of antitoxin expression.

**RecA-independent genomic exchange**

We modified the conjugal system to eliminate replication potential in the transferred segment in order to study lower frequency *recA*-independent recombination events. F’-Plasmid replication depends upon the RepE protein encoded in the 38 kb of proximal F DNA (between *oriT* and the clockwise chromosome junction) (S3 Fig.) [9]. The proximal F DNA is not required for DNA transfer during mating and even encodes two toxin-antitoxin systems that may promote plasmid retention (*ccdAB* and *flmAB*) [10,11]. Thus, we eliminated the entire proximal F DNA segment from the donor strain. For simplicity, this mutation is referred to as Δ*repE** (rather than Δ(*ygfA-ycaA*)::(FRT)).

When the resulting Δ*recA* Δ*repE** donor was mated with a *rec*^+^ recipient (Fig. 3 Cross 3), the recombination efficiency was the same as the efficiency observed in the analogous *repE*^+^ cross (Fig. 3, Cross 2) thereby confirming that the proximal F DNA deletion does not affect the donor’s ability to transfer DNA to the recipient.

Deletion of *repE* from the donor in the Δ*recA* mating background resulted in a ~17000 fold reduction in CFU/recipient per hour (Fig. 3, Cross 5 compared to Cross 4). 15-minute matings yielded no recombinants (data not shown), but a measurable recombination efficiency of (2.1 ± 0.2) X 10^-10^ CFU/recipient per hour was obtained after 18 hours of mating. Since conjugal DNA transfer is unaffected by the *repE* deletion (in the Rec^+^ background, compare Cross 2 to Cross 3), we attribute this extreme reduction in recombination efficiency to the inability to form F’-plasmids in the recipient. 90% of the recombinants from Cross 5 were KnS. Thus, the majority of recombinants derived from this configuration of the mating system derive from RecA-independent recombination events that replace the recipient’s ICR with that of the donor. The detailed nature of these events and of the less frequent KnR recombinants was discussed above.

**References**

1. Ryder L, Whitby MC, Lloyd RG (1994) Mutation of recF, recJ, recO, recQ, or recR improves Hfr recombination in resolvase-deficient ruv recG strains of Escherichia coli. J Bacteriol 176: 1570-1577.

2. Dermic D (2006) Functions of multiple exonucleases are essential for cell viability, DNA repair and homologous recombination in recD mutants of Escherichia coli. Genetics 172: 2057-2069.

3. Denamur E, Lecointre G, Darlu P, Tenaillon O, Acquaviva C, et al. (2000) Evolutionary implications of the frequent horizontal transfer of mismatch repair genes. Cell 103: 711-721.

4. Low B (1968) Formation of merodiploids in matings with a class of Rec- recipient strains of Escherichia coli K12. Proc Natl Acad Sci U S A 60: 160-167.

5. Chayot R, Montagne B, Mazel D, Ricchetti M (2010) An end-joining repair mechanism in Escherichia coli. Proc Natl Acad Sci U S A 107: 2141-2146.

6. Hastings PJ, Lupski JR, Rosenberg SM, Ira G (2009) Mechanisms of change in gene copy number. Nat Rev Genet 10: 551-564.

7. Low KB (1972) Escherichia coli K-12 F-prime factors, old and new. Bacteriol Rev 36: 587-607.

8. Hadley RG, Deonier RC (1980) Specificity in the formation of delta tra F-prime plasmids. J Bacteriol 143: 680-692.

9. Masson L, Ray DS (1986) Mechanism of autonomous control of the Escherichia coli F plasmid: different complexes of the initiator/repressor protein are bound to its operator and to an F plasmid replication origin. Nucleic Acids Res 14: 5693-5711.

10. Pedersen K, Gerdes K (1999) Multiple hok genes on the chromosome of Escherichia coli. Mol Microbiol 32: 1090-1102.

11. Hayes F (2003) Toxins-antitoxins: plasmid maintenance, programmed cell death, and cell cycle arrest. Science 301: 1496-1499.
