# Supplementary material for: Novel recA-Independent Horizontal Gene Transfer in Escherichia coli K-12
Source: PLoS One. 2015 Jul 10;10(7):e0130813. doi: 10.1371/journal.pone.0130813 (PMC4498929; doi:10.1371/journal.pone.0130813)
Supplement: S3 File — (DOCX) [file pone.0130813.s013.docx]

**Calculating the expected frequency of ICR-specific replacement**

The assumptions and calculations used to determine the expected frequency ICR-specific replacement are described here. This localized replacement event requires that both the proximal and distal crossovers occur in the intervals containing the borders of the ICR. If we assume that crossovers occur randomly throughout the genome, we can calculate the expected frequencies of simultaneous crossovers in these locations. If we constrain the crossover to the 90 kb between *oriT* and *mrr*::*tetRA* we would expect a random proximal crossover to occur within the 2.5 kb between *yjiP*::*cat* and *yjiT*::*npt* ~2.7% of the time (2.5/90). Genomic analysis of recombinant ER3466 shows that crossovers proximal to *oriT* can occur, so our calculations are a conservative overestimate of the probability of crossover to the left of the ICR. On the distal end, ~2.4 Mb separate *tetRA* from the nearest resistance marker (*gyrA96*), while the crossover interval that includes the ICR border spans 13.1 kb, putting the probability of a random crossover at 0.56% (13/2300). The probability of both of these events occurring at the same time in a recombinant is one in ~6600 (or ~0.015 %). Our matings produced recombinants with this crossover pattern at a much higher frequency (3/217 or 1.4%, nearly a 100-fold over-representation) (Fig 6). Thus, we obtain ICR replacement events at a frequency of 1.4% of all recombinants or roughly 2 X 10^-10^ CFU/recipient.
